# Development and *in silico* evaluation of large-scale metabolite identification methods using functional group detection for metabolomics

**DOI:** 10.3389/fgene.2014.00237

**Published:** 2014-07-28

**Authors:** Joshua M. Mitchell, Teresa W.-M. Fan, Andrew N. Lane, Hunter N. B. Moseley

**Affiliations:** Department of Molecular and Cellular Biochemistry, Markey Cancer Center, University of KentuckyLexington, KY, USA

**Keywords:** metabolomics, chemical adduct, chemoselection, Fourier transform mass spectrometry, isotope-edited NMR, common subgraph isomorphism, graph theory, functional group resolved metabolite databases

## Abstract

Large-scale identification of metabolites is key to elucidating and modeling metabolism at the systems level. Advances in metabolomics technologies, particularly ultra-high resolution mass spectrometry (MS) enable comprehensive and rapid analysis of metabolites. However, a significant barrier to meaningful data interpretation is the identification of a wide range of metabolites including unknowns and the determination of their role(s) in various metabolic networks. Chemoselective (CS) probes to tag metabolite functional groups combined with high mass accuracy provide additional structural constraints for metabolite identification and quantification. We have developed a novel algorithm, Chemically Aware Substructure Search (CASS) that efficiently detects functional groups within existing metabolite databases, allowing for combined molecular formula and functional group (from CS tagging) queries to aid in metabolite identification without *a priori* knowledge. Analysis of the isomeric compounds in both Human Metabolome Database (HMDB) and KEGG Ligand demonstrated a high percentage of isomeric molecular formulae (43 and 28%, respectively), indicating the necessity for techniques such as CS-tagging. Furthermore, these two databases have only moderate overlap in molecular formulae. Thus, it is prudent to use multiple databases in metabolite assignment, since each major metabolite database represents different portions of metabolism within the biosphere. *In silico* analysis of various CS-tagging strategies under different conditions for adduct formation demonstrate that combined FT-MS derived molecular formulae and CS-tagging can uniquely identify up to 71% of KEGG and 37% of the combined KEGG/HMDB database vs. 41 and 17%, respectively without adduct formation. This difference between database isomer disambiguation highlights the strength of CS-tagging for non-lipid metabolite identification. However, unique identification of complex lipids still needs additional information.

## Introduction

Metabolomics is the comprehensive study of metabolomes, which comprise the entirety of metabolites interconverted by networks of chemical reactions in living systems that make life possible and can be regarded as the functional readout of the genome and proteome (Kaddurah-Daouk et al., 2008; Le et al., 2012). Most of these chemical reactions are catalyzed by protein enzymes that interconvert a vast array of metabolites in complex networks.

Metabolites are bioorganic compounds that range widely in size and chemical complexity from small compounds with a few atoms (e.g., glycerol, C_3_H_8_O_3_) to more complex structures consisting of hundreds of atoms and multiple functionalities (e.g., monosialotetrahexosyl ganglioside C_77_H_139_N_3_O_31_). The ability to identify and quantify a wide range of metabolites is the first step in a systematic elucidation and modeling of metabolic networks. The next important step is the ability to track individual atoms of various metabolites through the metabolic network using isotopically enriched tracers (e.g., ^13^C, ^15^N, and/or ^2^H labeled precursors) coupled with stable isotope-resolved metabolomics (SIRM), from which metabolic networks can be robustly reconstructed (Fan et al., 2009, 2010, 2011, 2012; Moseley et al., 2011; Le et al., 2012). From such studies, we can acquire system biochemical insights across a broad spectrum of biological and biomedical problems (Lane et al., 2011; Ramautar et al., 2013; Armitage and Barbas, 2014; Wood, 2014; Zhang et al., 2014).

Despite the increasing interest in studying the metabolomes of different organisms, the systematic detection, identification, and quantification of metabolites, i.e., metabolomics, remains a challenge, which limits meaningful interpretation of metabolic data. Metabolomics employs numerous analytical techniques for elucidating metabolite structures and quantification, principally mass spectrometry (MS), and nuclear magnetic resonance (NMR). These complementary structure-based techniques afford a wider coverage of metabolites and versatility of structure determination, particularly in terms of isotopic enrichment patterns of metabolites in SIRM studies. For example, NMR is excellently suited for determining different position(s) of ^13^C label(s) in given metabolites (i.e., isotopomers) whereas MS readily provides the number of ^13^C atoms in a metabolite (i.e., isotopologs). Both types of structural information are required for robust reconstruction of metabolic pathways (Fan et al., 2012). The combination of NMR with high resolution high sensitivity FT-MS makes it possible to obtain molecular formulae of a large number of metabolites as well as isotopomer and isotopolog distributions (Pan and Raftery, 2007; Fan and Lane, 2008; Lane et al., 2008; Fan et al., 2012; Lorkiewicz et al., 2012).

The high volume of data produced by these instruments requires computational approaches for automated assignment of the spectra and to analyze the data in an accurate, meaningful, and timely fashion (Goodacre et al., 2004). Furthermore, the exceptionally high resolution and sensitivity of FT-MS allows for the detection of metabolites that have not yet been characterized, complicating peak assignment and analysis (Kind and Fiehn, 2006). Despite the extremely high resolution and mass accuracy of ultra-high resolution mass spectrometers, assigning a unique formula to most peaks remains a non-trivial problem. Only by utilizing isotope abundance and isotopolog data, which eliminates >95% of possible peak-formula mappings, can assignment of a peak to a unique formula or a small set of formulae be achieved (Kind and Fiehn, 2006). However, this approach fails when dealing with isotopically enriched metabolites in SIRM studies, where the natural abundance distribution no longer holds. The many more detectable mass isotopologs arising from each labeled metabolite demand even higher mass resolution and accuracy for isotope-resolved molecular formula determination, thereby making the existing assignment algorithms error-prone.

An equally difficult problem arises when the molecular formulae must be mapped to specific metabolites. This is typically done by referencing a database of interest and searching for entries that match the computed mass and/or formula for the mass peak of interest. For human metabolomics research, the Human Metabolome Database (HMDB) is a growing source for human-specific metabolite data (Wishart et al., 2009, 2013). The HMDB currently contains 40,427 entries for compounds observed in the human metabolome. In addition to the HMDB, the KEGG Ligand database also contains a large number of metabolite entries. Although not uniquely focused on human metabolism, the KEGG database currently contains 16,396 metabolic entries from a variety of species (Goto et al., 2002) and additionally numerous drug compound entries. The compounds from other species not yet observed in humans may provide possible hints as to the identity of observed, uncharacterized human metabolites or metabolites present in human tissue that derive, from external sources, like essential amino acids, sucrose, bacterial and plant products. For both databases, the entries are stored as variants of the MDL Molfile (.mol) format, a standard format for storing the chemical structure, atoms, bonds, ionization state, and stereochemical information needed to represent any given molecule (Dalby et al., 1992). However, database searching is ambiguous, as often any given formula can correspond to more than one entry. For example, using the MOLGEN isomer generator and the formula C_15_H_12_O_7_, 788,000 distinct structures are generated (even with restrictions on allowed functional groups) (Benecke et al., 1995; Kind and Fiehn, 2006). Fortunately, MOLGEN represents all *possible* structures, not just those that exist in known metabolic networks. Nevertheless, the presence of isomers, known as mass isomers in MS, greatly complicates the use of metabolite databases for metabolite assignment by MS. To overcome this difficulty, additional information must be obtained to accurately assign metabolite mass spectra. Tandem MS is often used to obtain chemical substructure of a given metabolite via its fragmentation pattern. Unfortunately, the data produced by tandem-MS requires very complicated, predictive algorithms for metabolite assignment and differences in fragmentation patterns generated by different instruments, in algorithms used for data analysis, and in data interpretation hampers the reproducibility and accuracy of these methods (Nesvizskii et al., 2007).

**Figure 1:**
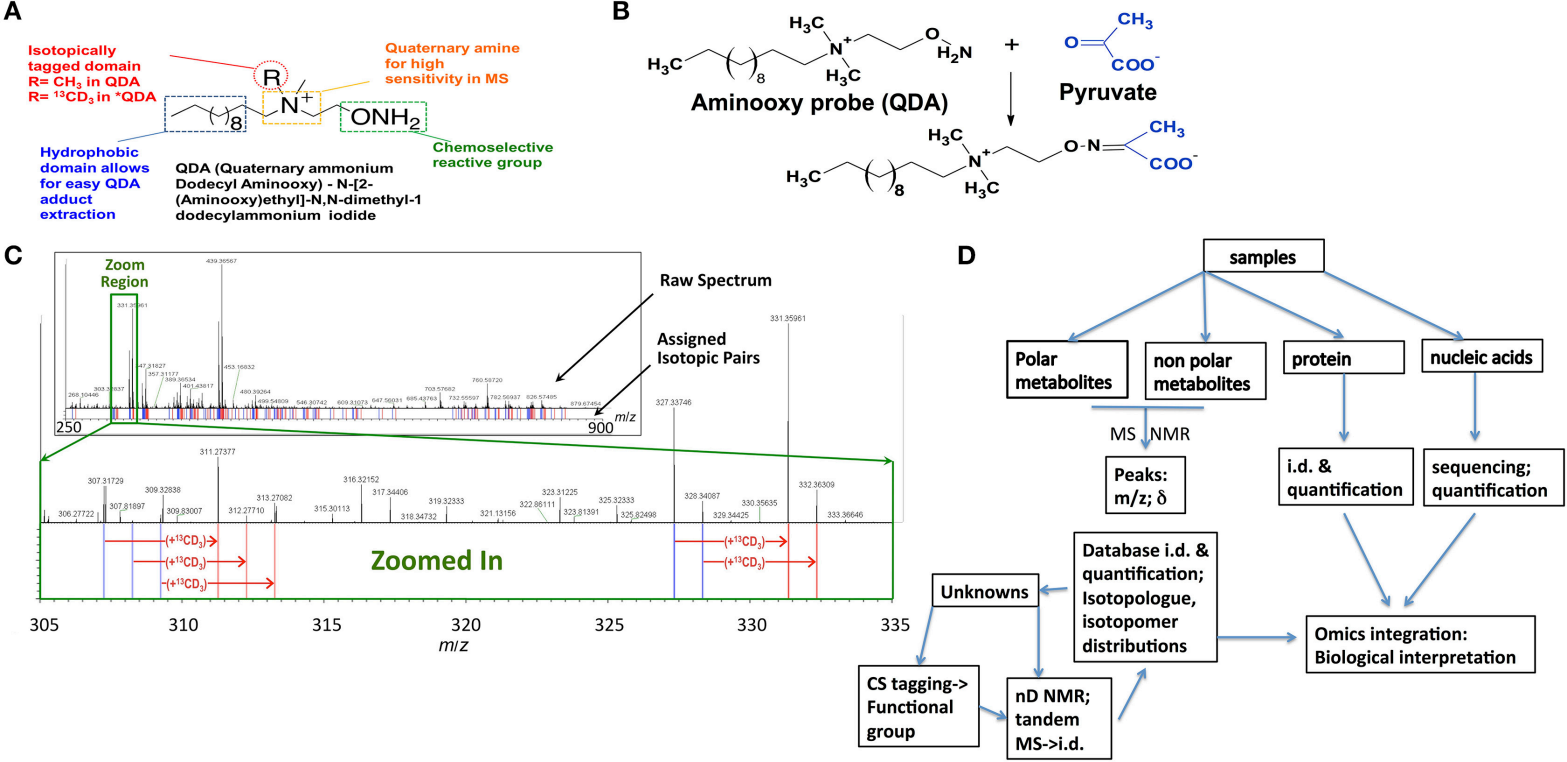
**Chemoselective tagging and large-scale detection of carbonyl-containing metabolites by FT-ICR-MS. (A)** A chemoselective (CS) probe (QDA) for tagging carbonyl-containing compounds was designed to contain three different functionalities, i.e., an aminooxy group for specific reaction with carbonyls, a quaternary ammonium group for enhancing MS detection, and a hydrophobic domain for partitioning CS adducts into organic solvents to remove ionic matrix interferences. In addition, the R group in QDA can be isotopically labeled (such as ^13^CD_3_ in ^*^QDA) to facilitate automated assignment of carbonylated metabolites (Mattingly et al., 2012). **(B)** Reaction between pyruvate and QDA to form the oxime ether adduct is depicted. **(C)** A crude polar extract of human lung adenocarcinoma A549 cells was reacted with an equal mixture of QDA and ^*^QDA before analysis by FT-ICR-MS. The top FT-ICR-MS spectrum shows an expanded spectral region from *m*/*z* 250 to 900, while the inset below (as depicted by the green box) more clearly illustrates the companion peaks of QDA and ^*^QDA adducts. Blue and red lines (below the spectrum) respectively denote the *m*/*z* values of unlabeled and ^13^CD_3_-labeled QDA derivatives, giving a “bar-code” profile of the adduct pairs (adapted from Mattingly et al., 2012). **(D)** Flow diagram from sample through to data integration. Samples from various sources are extracted into separate fractions of polar and non-polar metabolites and proteins (single step, Fan, 2012) and nucleic acids, either genomic DNA or mRNA (Fan et al., 2012). The metabolites are either separated by chromatography for MS (e.g., GC-MS, LC-MS) or subjected to direct infusion high resolution MS and NMR (Lane et al., 2008, 2009). The knowns are identified by comparison with standard databases (Fan and Lane, 2008; Lane et al., 2008). The unknowns may be identified using additional experiments including tandem MS and multidimensional NMR, especially if isotope enriched and where editing techniques can be used. Alternatively, the samples can be reacted with functional-group-specific reagents that introduce a tag that can be edited for by NMR, and imparts increased sensitivity in MS. This additional information is used to narrow down the possibilities in the database searches as described in the text.

Chemoselective adduct formation, i.e., CS-tagging, of metabolite functional groups, with subsequent detection by ultra-high resolution FT-MS and/or NMR provides additional sources of chemical structure information that could facilitate the unique assignment of metabolites. Isotopically enriched reagents can be designed to react with particular functional groups present in metabolites, such as carboxylate (Ye et al., 2009), carbonyl (Fu et al., 2011; Mattingly et al., 2012), amino (Guo and Li, 2009), and sulfhydryl (Gori et al., 2014). Figure 1A shows the carbonyl-selective aminooxy reagent for simultaneous MS and NMR chemical editing. The adducts formed can be selected by isotope editing techniques by NMR or in high resolution MS, and the tag further provides enhanced sensitivity for MS (cf. Figure 1B). The subset of metabolites that react must therefore contain the particular functional group (Figure 1C), which when combined with stable isotope labeling of the aminooxy reagent and detection by high mass accuracy and isotope edited NMR shift data can often identify the metabolites uniquely, especially resolving isomeric structures (cf. workflow in Figure 1D). This CS-tagging approach provides information that directly relates to chemical substructure, and can be combined with accurate mass and fragmentation patterns from tandem-MS methods. However, in order to efficiently use functional group composition information along with molecular formulae, metabolite databases with functional groups delineated are needed.

Identifying functional groups in existing metabolite databases provides a convenient way of creating such a functional group-resolved metabolite database. Fundamentally, this problem requires the identification of metabolite substructures that are identical to functional groups of interest and storing this information in a well-organized manner as part of each metabolite entry. CheckMol is a publically available program which can determine the presence and number of over 240 different functional groups in molfile files (Haider, 2010b). Since its introduction in 2003, CheckMol has remained the industry standard for detecting functional groups within chemical structures and is a component in several chemoinformatics packages. Although CheckMol is a powerful and reliable tool, it does not use a generalized method for searching for each functional group; rather the method used for each functional group is unique and hard-coded. In order to add a new functional group to the list of functional groups searched for by CheckMol, a new method must be written in Pascal and then incorporated into the proper region in CheckMol, without introducing errors (Feldman et al., 2005).

To develop a tool that can search for a user-defined set of functional groups using a generalized strategy that does not require code modification, a natural choice is to abstract a molecule as a graph, in which the atoms are nodes and the bonds are vertices. The problem of detecting similarity between structures then is analogous to that of finding regions of similarity between the two graphs, called isomorphisms. This is the well-documented maximum common subgraph isomorphism (MCSI) problem in graph theory, for which several algorithms already exist, such as the Ullmann Algorithm (Ullmann, 1976). Also, graph theoretical approaches are widely used in chemoinformatics, notably to evaluate the structural similarity between compounds (Hattori et al., 2010) and to aid in the assignment of MS data (Hummel et al., 2010).

The Ullmann algorithm in its original form is unsuitable for our application as it implements a time-consuming brute force method for finding isomorphisms and lacks optimizations for isomorphism search in the context of chemical structures (Raymond and Willett, 2002). We have now implemented a novel algorithm loosely based on Ullmann's for finding subisomorphisms in database compounds that are completely isomorphic with a specific functional group. Our algorithm, called Chemically Aware Substructure Search (CASS), solves the subgraph isomorphism problem, which is NP-complete in computational complexity, but not NP-hard as in the case for MCSI. CASS utilizes a short-circuiting method to greatly accelerate the search for isomorphisms as well as a set of optimizations based on chemical structural rules. Although metabolite molfile files are readily available from KEGG and the HMDB, there is no database of functional group molfile files. We have hand crafted a database of 210 functional group molfile files using JChem which includes most of the functional groups searched for by CheckMol (Csizmadia, 2000). By applying our tools to both KEGG Compound and HMDB, we have constructed a functional group-resolved database that combines the two databases into SQLite (Owens, 2006) relational tables. This database can be queried using the formulae detected by FT-MS along with CS-tagging to aid in the assignment of metabolites. Furthermore, additional chemical substructure information derived from either MS-MS analysis or NMR can be readily incorporated into the analysis by simply adding additional substructure molfile files for query.

## Materials and methods

### Database access

Although both the HMDB and KEGG databases are publically accessible from web interfaces, local copies of the databases were needed for our analyses. The HMDB database was downloaded directly as a single SDfile (.sdf) file (i.e., flat file of concatenated molfile files with additional structured information) from the HMDB website. Like many sources of molfile files, the most recent versions of the HMDB contain additional structural and chemical information in each molfile file that is not specified in the original V3000 molfile file specification; therefore we developed a Perl script to handle these standard deviations from the molfile file specifications and create a specification compliant version. As the KEGG Ligand database is not available for download in any consolidated format, we developed a Python program that takes advantage of the KEGG REST interface to download molfile files (or kcf files) for each entry in the database and then concatenate them into a local copy of the KEGG database. The molfile files for KEGG entries do not contain database IDs nor compound names; these were collected from the KEGG database via its REST interface and added to the appropriate molfile file by our Python program.

**Figure 2:**
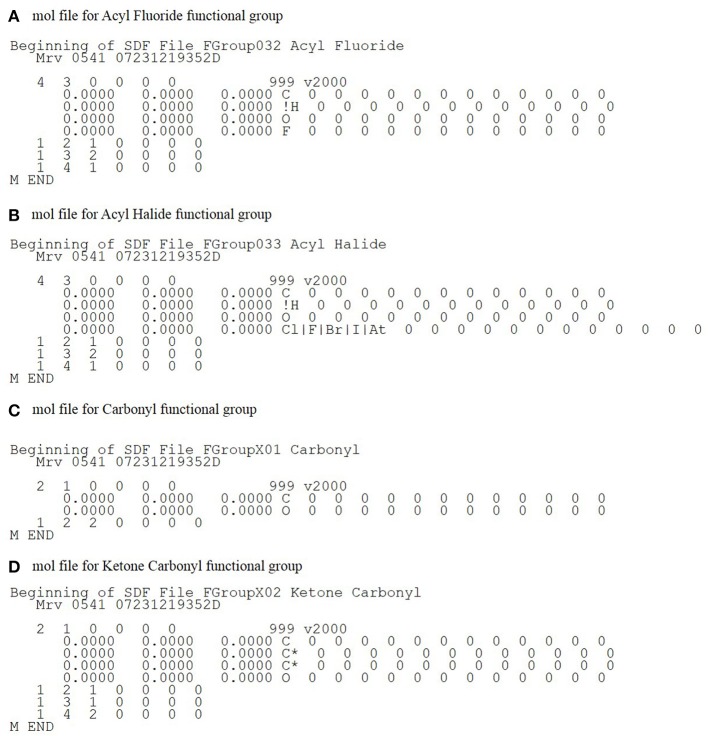
**Example functional group molfile files. (A)** The functional group molfile file for acyl fluoride demonstrates the use of the !X element type. The !H element type for atom 2 designates that it can be validly mapped to any non-hydrogen element type atom. **(B)** Similar to acyl fluoride, acyl halide uses the !H to designate a non-hydrogen element type. Additionally, since the halogen component of an acyl halide can be any halogen, the element type for the halogen atom is designated using the X|Y element type. **(C)** A typical functional molfile file. (**D)** The ketone carbonyl functional group uses contextual atoms to prevent matching of the molfile files to carbonyl-containing moieties that are not ketones.

Because we could not find a current functional group database that fit our particular design criteria (ability to specify both wild-carded and contextual atoms) (Kotera et al., 2008; Haider, 2010a,b; Eustis, 2011), we created one from scratch. To provide the same functionality as the existing CheckMol program, the list of functional groups detected by CheckMol was a natural starting point. For each functional group, the structure of the functional group was drawn by hand in JChem and the structures saved as molfile files. The molfile format designates each atom as a particular element. Therefore, we have developed a new nomenclature for describing these conditions. To designate that a particular atom could be one of several element types, the element type is designated as a list of possible element types separated by “|” while an “!” before an element type specifies the element type can be any element except the specified one (Figure 2). For example, “H|O|N” as an element type would specify that the atom could be hydrogen, oxygen or nitrogen while “!H” specifies that the element type can be any element type other than hydrogen. These descriptive facilities are more powerful than simple wild-carded “^*^” descriptive facilities available in other chemoinformatics tools (Daylight Chemical Information Systems, 2008).

Furthermore, to allow searching for a specific chemical substructure (e.g., −C=O or carbonyl) in particular chemical contexts (e.g., aldehydes or ketones), a way to designate atoms as “contextual” was added. Contextual atoms are designated with an asterisk after the element type and must be matched for a chemical substructure but are not considered as part of the substructure. For example, “C^*^” indicates a required element type of carbon that is not counted as part of the chemical substructure (Figure 2), for example, to distinguish between ketone and aldehyde carbonyls. To identify ketone carbonyls exclusively, the two carbon atoms bonded to the ketone carbonyl carbon atom are designated as contextual and therefore must be matched for the ketone carbonyl to be recognized but are not considered as part of the ketone carbonyl substructure. As a result, the carbonyl of an aldehyde, which is bonded to C and H, would not be recognized. The ability to designate contextual atoms in our chemical substructure descriptions is one of the main differences from previously published chemoinformatics toolkits that have substructure detection facilities. For example, while SMARTS allows for wild-carded atom designation (Daylight Chemical Information Systems, 2008) it does not allow for the designation of contextual atoms. This ability allows CASS to cleanly determine which atoms overlap between functional groups.

The functional group molfile files were concatenated to form a flat database similar to the downloaded copies of KEGG and the HMDB. Since flat files themselves provide no efficient means of searching for a particular entry and therefore must be parsed in their entirety, SQLite versions of these flat database files were created, to enable indexed entry retrieval. SQLite retains the simplicity and portability of flat files while offering the ability to search for entries in an efficient manner. Additionally, tools written in Perl were created to add a new entry to a SQLite database from a molfile file, return a particular molfile file from a database, and to check if a given entry exists in the database.

### Molfile parsers

While molfile files accurately store chemical structures in a human-readable format, the structure of the molfile file format does not lend itself to computer manipulation and thus a more computer friendly internal format was needed. Toward this end, a molfile file parser was developed to convert molfile files into an internal representation shared among all of the programs. Due to differences between the formatting of KEGG and HMDB molfile files, different parsing methods are required for each database molfile file. Our parser can handle the molfile file variants used in both KEGG and HMDB as well as the proprietary.kcf format used in KEGG. This parser also handles the modified molfile file format used in our functional group molfile files via a parameter passed to the parser.

Regardless of the origin of the input molfile file, the final data structure generated by the parser is the same, a “molecule” object consisting of multiple data members representing different constituents and properties of a molecule. For each atom in the molfile file, an “atom” object data member is created that contains the element type, number of bonds to the atom, the sum of the bond order of all bonds to the atom and the index of the atom, which is its order in the list of atoms in the molfile file. Similarly, each bond has a corresponding “bond” object data member containing the indices of the two atoms it bonds and the order of the bond. Additionally, the molecule object contains the compound's database ID and name, a mathematical representation of its bonded structure, and optionally, a string representation of the molfile file from which it was generated.

In many database molfile files, implicit hydrogens are often excluded to reduce the size of the files. These implicit hydrogens must be added to the internal representation of each compound as the hydrogens could be included in a functional group of interest. We used standard molecular connectivity and valence methods to add the missing hydrogens (Weininger, 1988). This procedure does not account for pH or pK in these calculations and hydrogens are added to produce non-charged molecules unless the molfile file specifies otherwise (i.e., species that barely exist in practice). This procedure was validated by comparing known formulae for database compounds to computed formulae following hydrogen addition. Owing to the deviation of KEGG and HMDB molfile files from the molfile file standard, preexisting packages for manipulating molfile files could not be used and our own tool had to be created. These new tools add a variety of features in addition to adding implicit hydrogens and they support non-standard molfile files and KEGG compound files (.kcf), a molfile file derived file format used throughout the KEGG database.

### Adjacency matrix representations

**Figure 3:**
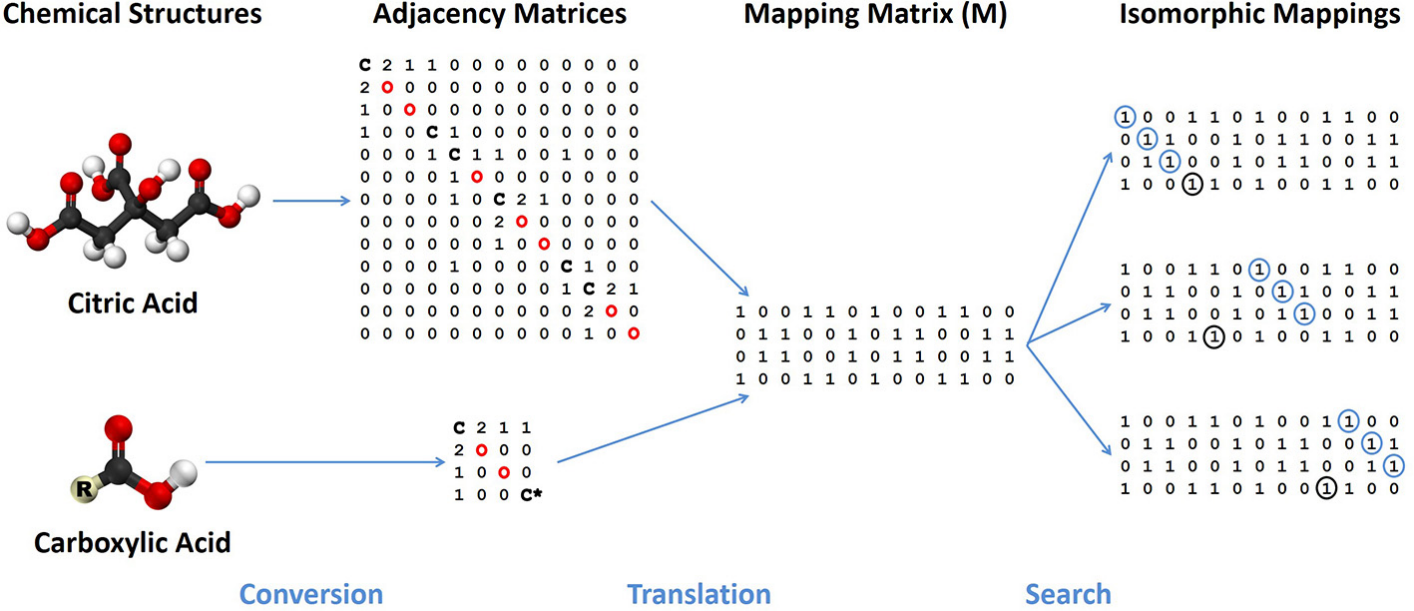
**Flowchart of major steps and representations needed by a chemical substructure search algorithm**. Chemical structures of a specific compound and functional group are treated as colored graphs that are converted into adjacency matrices with elements identified along the diagonal. A mapping matrix (**M**) represents possible translations of specific atoms between the compound and functional group. The mapping matrix is searched for specific isomorphic mappings of the functional group mapped completely onto the compound chemical structure.

In order to use the graph theory algorithms in our substructure search program, numerical representations of each database's chemical structure are needed. The two common options for storing graph-like structures are adjacency lists and adjacency matrices. Although the list representation requires less memory than an adjacency matrix, matrices allow for direct testing of isomorphisms using very quick matrix comparisons and multiplication. In an adjacency matrix, each row and column corresponds to a specific node in a graph, or in this instance, an atom in a molecule (see Figure 3). The assignment of row or column to atom is done using the index of the atom. Row and column N is mapped to the atom with index N, therefore the first row and column both represent the first atom, the second row and column the second atom and so on. The value for an element (*i,j*) of the adjacency matrix corresponds to the presence or absence of a vertex connecting the two nodes, which correspond to chemical bonds between atoms. If the value of *i,j* is zero, no bond exists between the atoms.

To construct an adjacency matrix for a molecule with N atoms, our program first creates an N × N square matrix (**A**) with all **A**_i,j_ equal to zero. This saves a significant amount of time constructing the matrix as the entire matrix object is initialized at once and memory is already allocated for it. Second, as molecular graphs are often sparse (i.e., the number of possible vertices is much smaller than the maximum possible number of vertices), most of the values of **A**_i,j_ will be equal to zero. Thirdly, such a matrix can be created very efficiently utilizing functional programming methods which are heavily optimized in Perl. Not all values of **A**_i,j_ can remain zero, so for each bond object, the indices of the bonded atoms are retrieved along with the bond order and the corresponding values of **A**_i,j_ and **A**_j,I_ (as bonds are mutual) are set equal to the bond order. For example after processing, a double bond between atoms 2 and 4, **A**_2,4_ = 2 and **A**_4,2_ = 2. Once the adjacency matrix is constructed, they are stored as an object data member within the molecule object.

### Substructure searching

**Figure 4:**
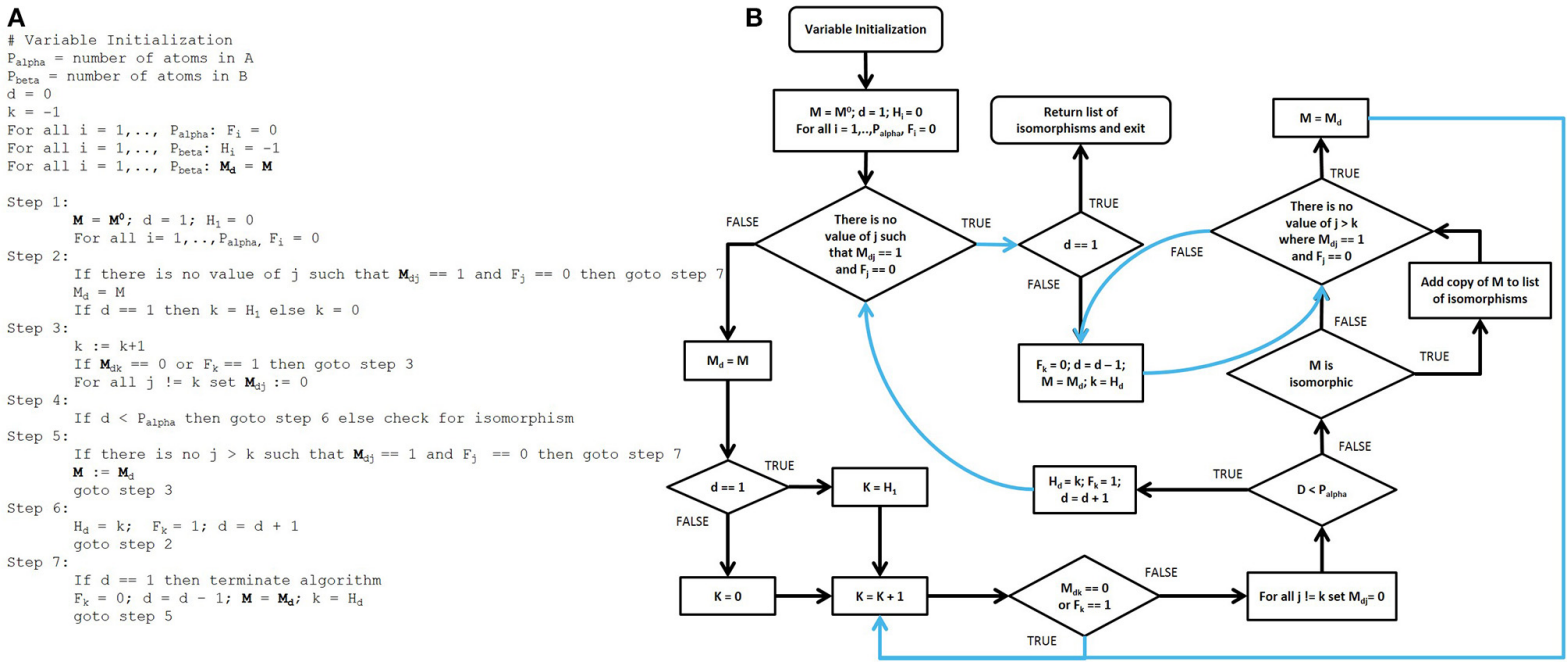
**Pseudocode and control flow diagram of the original Ullmann simple enumeration algorithm. (A)** The original pseudocode for the Ullmann simple enumeration algorithm. The symbol “:=” was used in the original algorithm to denote assignment (e.g., d := d + 1 means “set d equal to d + 1”). **(B)** Control flow diagram generated from the original published 7-step Ullmann enumeration algorithm pseudocode due to the presence of numerous “goto” statements. Although “goto” statements still exist in some programming language, their use are highly discouraged in modern programming style in order to prevent errors and to improve both the readability and the maintainability of computer programs. The “goto” statements are represented by blue lines in the control flow structure. The variable SKIP2B was added and maps to no variable in the original algorithm, it is for control flow purposes only.

**Figure 5:**
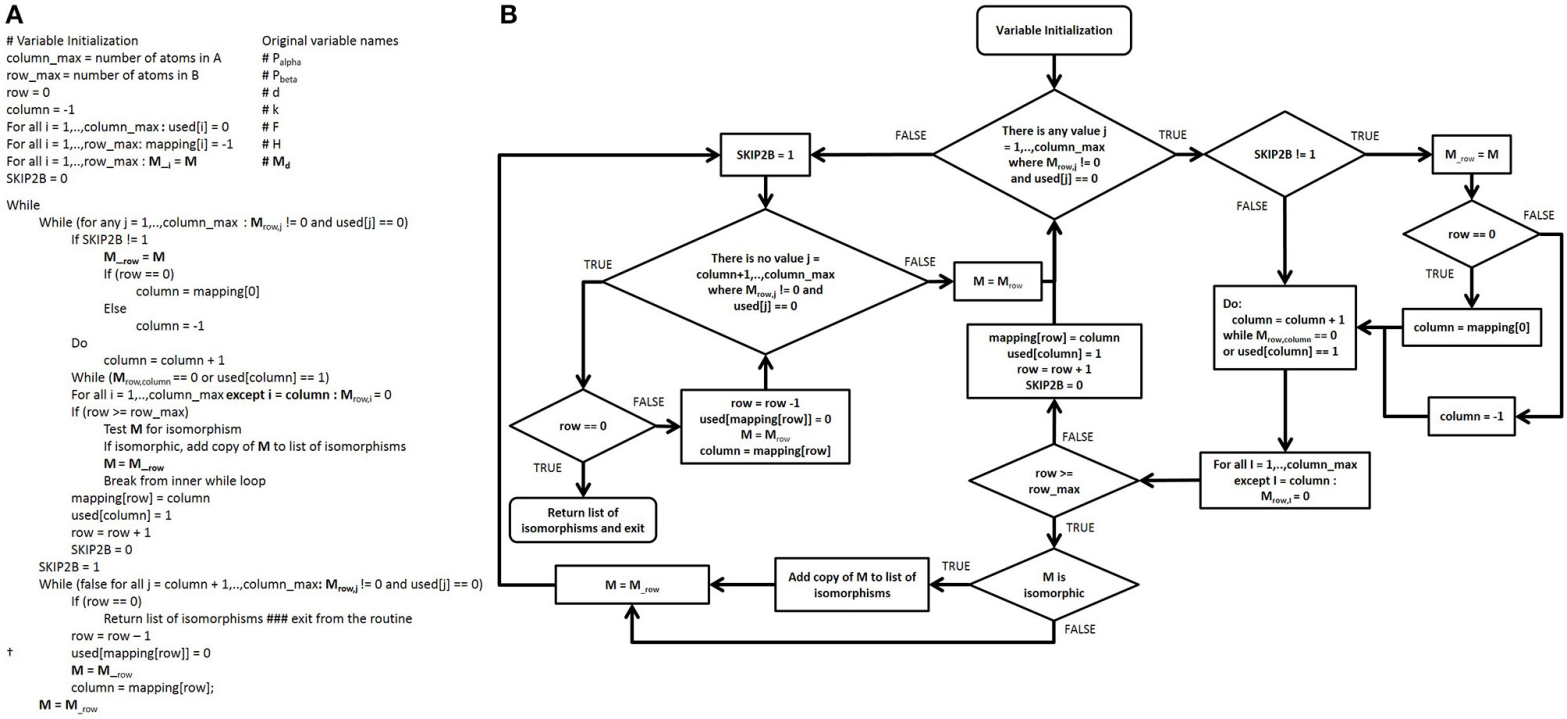
**Modernized pseudocode and control flow diagram of the Ullmann algorithm. (A)** Modern Ullmann algorithm pseudocode produced from the control flow diagram with variables renamed to promote readability of the pseudocode. While converting the control flow diagram to the pseudocode, we noticed a typographical error in original published algorithmic pseudocode (line 2, step 7, in the simple enumeration algorithm on page 33 of the original publication), which we assume has been either overlooked or ignored since its publication. This line is marked † in the pseudocode above. **(B)** Control flow diagram of the modernized Ullmann algorithm.

After the adjacency matrices for both the database compounds and the functional groups are constructed, our algorithm searches for isomorphic functional group substructures within the database compounds. The starting point for our algorithmic development was the Ullman algorithm (Ullmann, 1976). Owing to the presence of numerous “goto” statements in the original pseudocode, we converted this pseudo code into a control flow diagram (Figures 4A,B) and then into a modern control flow pseudocode representation (Figures 5A,B). We then deviated significantly from this new pseudocode representation during the development of our algorithm.

Given two graphs *G_A_* and *G_B_* representing the structure of a database molecule A and a functional group or a generic substructure query B and their corresponding adjacency matrices **A**_A_ and **A**_B_, the first step in both algorithms is the creation of a mapping matrix **M** (see Figure 3) with dimension b × a, where a and b are the number of atoms in A and B, respectively. It should be noted that a > b, as B must have fewer atoms than A, in order to be a subgraph of A. Each element of **M** is then assigned a value of 1 or 0. If **M**_i,j_ = 1, the atom with index i in B can be “validly mapped” to the atom with index j in A and if **M**_i,j_ = 0, no valid mapping can exist between the two atoms. In the traditional Ullmann algorithm, the definition of a valid mapping was determined by the number of vertices to the two nodes, i.e., valid mappings can only exist when the number of vertices to the jth point in A is greater than or equal to the degree of the ith point of B. Thus, the number of vertices “colors” the node and valid mappings are only allowed between nodes with the same or appropriate “color.” Expressed in chemical terms, the jth atom in A must have an equal or greater number of bonds as the ith atom in B. By expanding the parameters that constitute a valid mapping, the total number of possible mappings that have to be tested can be minimized. In our algorithm, the element types of the two atoms are compared as well, set the corresponding **M**_i,j_ equal to zero (**M**_i,j_ = 0), if the element types do not match. Here our expanded element types used in the functional group molfile files is important, as !X could map to an atom not element X (**M**_i,j_ = 1), X|Y|Z could map to an atom of element type X, Y, or Z (**M**_i,j_ = 1). As we are searching for complete instances of the functional group B as a substructure of A, every atom in B must have at least one valid potential mapping to an element in A. Therefore, if an entire row of **M** contains zeroes, no isomorphism can exist for that functional group-database compound pair as there is an atom with no possible valid mapping.

**Figure 6:**
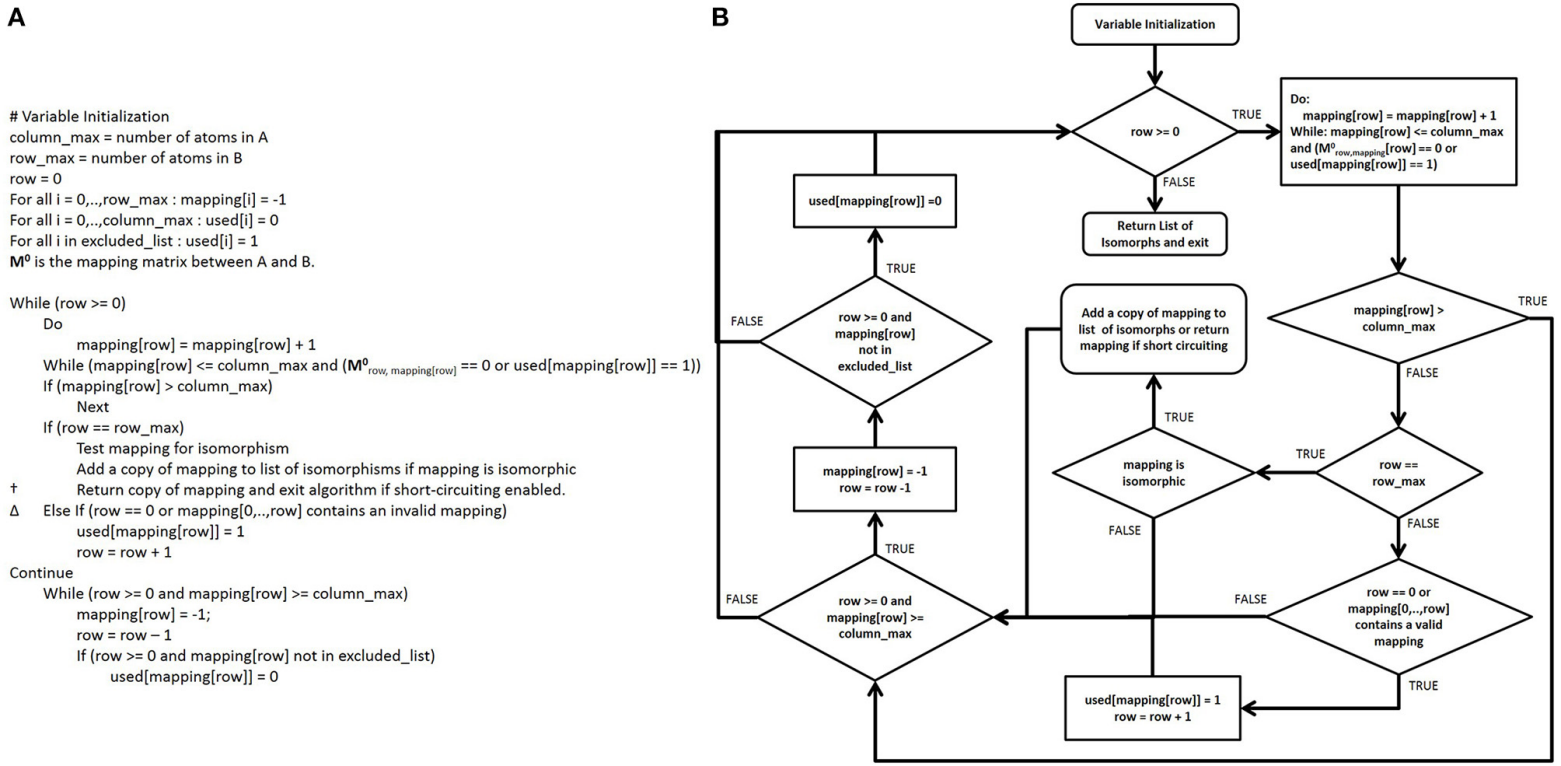
**Pseudocode and control flow diagram of the CASS algorithm. (A)** Pseudocode for CASS. In this algorithm, matrices are neither copied nor modified, saving considerable computational time and memory. Also, the control flow is cleaner than in the modernized Ullmann algorithm pseudocode. Additionally, our short-circuiting method in the line marked † allows the algorithm to terminate early when a valid mapping has been identified. This allows for time savings when knowing that a single valid mapping exists is sufficient and the number of valid mappings is not needed (e.g., stereoisomerism detection). Also, partial invalid mappings allow additional short-circuiting to take place in the line marked Δ, since the algorithm is finding subgraphs in graph A that are isomorphic to graph B. **(B)** A control flow diagram of CASS.

**Figure 7:**
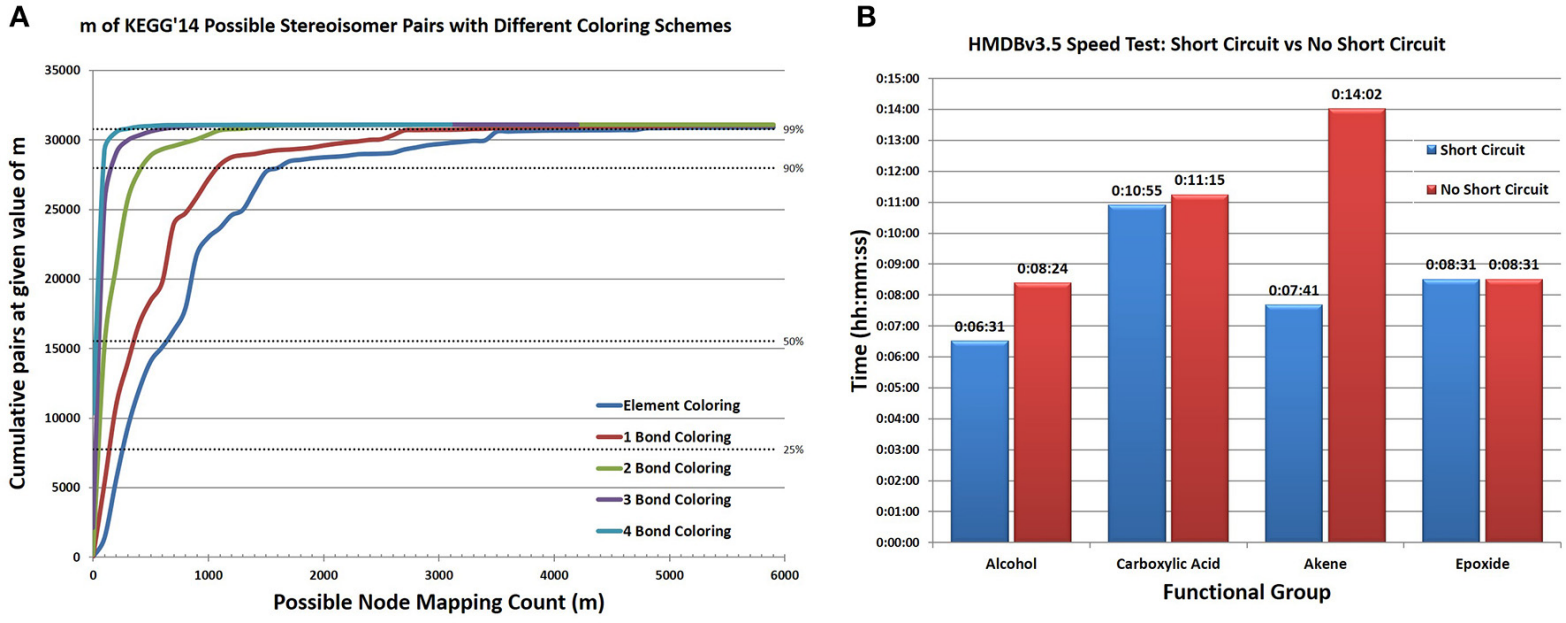
**Representative results of significant algorithmic improvements in CASS. (A)** With element coloring only, the average number of mappings for each possible stereoisomer pair ranges from very small to very large, with 50% of pairs having an *m* greater than 700 and a maximum *m* of 25,219 (not shown in figure). Coloring using the element types connected within N bonds reduces *m* for almost all pairs. With 1 Bond coloring, 50% have an *m* greater than 400. A substantial improvement occurs between 1 bond and 2 bond coloring, with 50% having an *m* greater than 100 and a maximum *m* less than 6300. Incremental improvements in *m* occur for most compounds when *N* > 2. Using coloring schemes based on adjacent atoms greatly improves the speed of finding possible stereoisomerism with a negligible time investment to color each atom. This allows CASS to efficiently check for stereoisomerism between very large structures. **(B)** By searching for the four functional groups in the entirety of the HMDBv3.5 the time savings observed by enabling short circuiting mirrors the improvement seen in our trial set. Alcohols and alkenes show a large improvement while epoxides and carboxylic acids do not. This likely relates to the relative frequency of the functional groups. Very common functional groups show the greatest improvement as it is very likely to find an instance of the group early in the enumeration.

Since **M** represents simultaneously all possible mappings, not individual mappings of functional group atoms to database compound atoms, **M** must be searched to find specific mapping matrices **M'** for each mapping of all functional group atoms to particular database atoms (Figure 3). Thus, a comprehensive search of **M** enumerates all **M'** and the computational speed of searching **M** is highly correlated to the number of “1” elements in **M**, which we call the “possible node mapping count” (*m* = ∑ **M**_i,j_). Now, the Ullmann algorithm directly searches **M** in a depth-first manner; this involves copying and modifying large two-dimensional matrices frequently to enumerate all **M'**. Our algorithm avoids these costly operations by keeping track of the enumeration process with two one-dimensional integer vectors, **v** and **u**. |**v**| is equal to the number of atoms in B and **v** records which atoms in B are mapped to atoms in A at any stage of the enumeration. The index of the element in **v** corresponds to the index of the atom of B and the value of **v**[*i*] the index of the atom in A to which it is mapped; so the value **v**[2] = 3 denotes that atom two in B is currently mapped to atom three in A. Before any value **v**[*i*] = *j* is assigned, we check that **M**_i,j_ = 1, so that the mapping stored in **v** is potentially valid. To denote an unmatched atom in B, the corresponding element of **v** is set equal to −1. Since **v** stores the same information as **M'** in the Ullmann algorithm, we can skip explicitly calculating **M'** all together saving both time and memory. In circumstances where knowing the existence of a valid mapping is sufficient, once a valid mapping is detected the algorithm can return the valid mapping and terminate. When applicable, this short-circuiting has the potential to substantially improve performance when the number of possible valid mappings is very large or the likelihood of finding a valid mapping early in the enumeration process is high (see **Figure 7A**). |**u**| is equal to the number of atoms in A and the elements in **u** indicate if a corresponding atom in A has been used in previously detected valid isomorphisms and should therefore be excluded from further enumeration. The index of a value in **u** represents the atom with the same index in A and the value of **u**[*i*] is either zero or one, representing if the column is non-excluded or excluded, respectively. The pseudocode for our enumeration method is shown in Figure 6.

Each **M'** generated by the Ullmann algorithm contains only one “1” per row and represents a particular mapping of the atoms, which must be checked to confirm if it is a valid isomorphism. The Ullmann algorithm checks for isomorphism by comparing a matrix **C** to **A_B_,** where **C** = **M'**(**M'A_A_**)^T^., An isomorphism is found if it is true that (∀*i,* ∀*j)* where (**A**_Bi,j_ = 1) then (**C**_i,j_ = 1). In our algorithm, we circumvent the calculation of **C** by directly comparing **A**_A_ and **A**_B_ using the information stored in **v**. If (∀ 0 ≤ *i* ≤ |**v**|, ∀ 0 ≤ *j* ≤ |**v**|) (**A**_B i,j_ = **A**_Av[i],v[j]_), then **v** represents a valid isomorphism and a copy of **v** denoted as **v'** is stored in a list of isomorphisms. Once an atom in the functional group has been discovered in a valid isomorphism, the corresponding element in **u** is set to one to exclude that atom from additional enumeration. Additionally, values in **u** can be given as input to the enumerator to prevent mappings to those atoms. This is useful in excluding database compound atoms from searches or to import information concerning previously detected chemical substructure.

After all functional group-database compound pairs are checked for potential isomorphisms, it must be determined if these isomorphisms overlap one another or are subgraphs of one another. These conditions can be determined quickly by comparing the saved **v'** from each identified isomorphism. Consider two functional group isomorphisms E and F and their corresponding mapping vectors **v**_E_ and **v**_F_. First corresponding sets are constructed from each vector and the values with indices corresponding to context-only atoms are removed: V_E_ = {**v**_E_[*i*]|*i* is not the index of a context only atom in E} and V_F_ = {**v**_F_[*i*]|*i* is not the index of a context only atom in F}. With these sets constructed, the vertices shared by E and F is simply the set O = V_E_ ∩ V_F_ and the relationship between E and F can be determined by comparing O to V_E_ and V_F_. If O = Ø, the two sets are disjoint and therefore E and F do not overlap. If |O| = |V_E_| = |V_F_|, the indices shared are identical and E and F represent mirror images of the same substructure. If |O| = |V_E_ | and O != Ø then E completely overlaps with F and E is a subgraph of F. If |O| = |V_F_ | and O != Ø then F completely overlaps with and is a subgraph of E. Else, |O| < |V_F_ | and |O| < |V_E_ | and O != Ø E and F overlap but neither is a subgraph of the other. This allows the program to differentiate functional groups that exist as a subgraph of other functional groups from those that do not and allow for proper counting of functional groups that are mirror images. Functional groups that are determined to be a subgraph of another functional group (conditions 2 and 3) have “subgraph” appended to their name. Functional groups that are overlapping but neither is a subgraph of the other (condition 4), both functional groups have “overlapping” appended to their name. For example, the hydroxyl group of a carboxylic acid would be designated a “subgraph-hydroxyl” while the carboxylic acid would be designated simply as “carboxylic acid.” Additionally mirror image functional groups such as anhydrides, match twice, and this must be accounted for in order to arrive at the proper number of instances of such substructures. This comparison is conducted for all functional group pairings and once complete, the name and number of functional groups is appended to the molecular formula to generate an “extended formula.” For example, if only ketones were searched for, the extended formula for acetone would be C_3_H_6_O_1_Ketone_1_. Functional groups can also be marked as “super” functional groups. These groups are excluded from the subgraph and inclusive designations and are used for functional groups that match a large number of other functional groups in the database or are a subgraph of many other functional groups. Alkyl halide is such a “super” functional group as it matches alkyl chloride, fluoride, iodide, and bromide; if not marked super, all instances of alkyl chloride for instances would be overlapping with alkyl halide.

In addition to searching for functional groups, CASS can also be configured to search for potential stereoisomerism between database compounds. First, all database compound pairings between database entries with the same molecular formula or extended molecular formula are identified. Searching by extended formula can greatly decrease the number of non-stereoisomeric pairing that must be tested as stereoisomers will contain the same functional groups in addition to having the same formula while other types of isomers may not. When searching for stereoisomers the same process as used for functional groups is utilized except that compounds A and B are the database compounds being tested. As our adjacency matrices do not store stereochemical information and oftentimes database molfile files only have 2 dimensional coordinates for the atoms, we do not utilize 3 dimensional coordinates in making this analysis, only the knowledge that two compounds have the same connectivity between their atoms. Implicit and explicit hydrogens can be omitted during this search to improve performance, since confirming two structures as stereoisomorphic is very time consuming, especially for large molecular graphs, where a large number of “bad mappings” must to be tested. Therefore, we had to expand our “node coloring” scheme. Thus, we included the “color” of bonded atoms to create a complex “patterned color” for an atom. This scheme can be recursively applied to include larger shells of bonded atoms. We refer to our initial coloring scheme as “element coloring” and then each shell of atoms included as “1-bond coloring,” “2-bond coloring,” etc. This improved node coloring scheme greatly reduces the size of *m* (Figure 7A), making detection of stereoisomers of large compounds tractable. Still, duplicate entries or duplicate structures cannot be distinguished using this method; although, it is reasonable to assume that the percentage of duplicate entries within any one database is very small and that stereoisomers identified by this method represent true stereoisomers. While this advanced node coloring scheme is straightforward to apply for stereoisomer analysis, it is harder to apply to functional group searching, due to boundary conditions for nodes with edges outside the functional group.

### Storing functional group information

Although CASS finds functional groups in relatively short time, it is undesirable to repeat the calculations every time the data needs to be accessed. To prevent repeating costly calculations, the functional group data for the database entries is stored as a SQLite database. All of the database entries are stored in one table with their molecular formulae, extended formulae, molfile files, text representations of the atom and bond objects and the number of each functional group present, including separate entries for overlapping and subgraph functional groups. Additionally the functional group molfile files and a list of the functional group names used when the database was created is stored as a separate table in the SQLite database. This is to allow for maximum portability and flexibility as everything needed to add a new compound entry is available in the SQLite database. With the appropriate program, a pre-existing functional group resolved database, and the molfile file for a new compound entry, the additional entry can be added with the functional groups stored in the SQLite database without reconstructing the entire database. However, if the list of functional groups is changed, the database must be reconstructed as the number of overlapping and subgraph functional groups may change. This SQLite format allows rapid and efficient searching for database compounds with certain properties including molecular formula and/or functional group composition, matching our standard use-case involving such information derived from CS-tagging and acquired by FT-MS.

### CS-tagging strategy analysis

After using CASS to determine the number of each functional group within all database entries, the functional group identified databases were analyzed to determine which combinations of functional groups under what conditions allows for the best disambiguation of isomeric database compounds. A specific CS-tagging strategy is represented by a set of functional group adducts and its' “performance” is measured by the number of non-isomeric extended formulae obtained from the database using the percent of non-isomeric compounds from the combined database as the base line.

As the number of functional groups in our functional group database is too large to test all permutations of all possible functional groups, strategies were generated iteratively assuming that functional group inclusion will have an additive effect on strategy performance. Therefore, strategies performing above a certain cutoff are expanded to include an additional functional group while poorly performing strategies are eliminated. In the first iteration, all one-functional group strategies are generated and the top 50 best performing strategies kept. For all iterations *i* > 1, the strategies from *i* − 1 are expanded to generate all pairings of each parent strategy with each functional group detected in the database to generate new child strategies. The performance of each child strategy is compared to the performance of the parent strategy; if the performance difference does not exceed a user-specified limit, the child strategy is removed. The top Y best performing non-redundant child strategies are then kept and passed into the next iteration. This process continues until the specified number of iterations is met or until an iteration generates no new child strategies above the performance cutoff.

As two functional groups (A and B) can perform synergistically, where in strategy [A,B] provides a greater disambiguation of isomeric compounds than the performance of [A] plus the performance of [B] would predict. Therefore, for effective strategy searching X and Y must be sufficiently large to allow poor performing strategies a chance to be paired with a synergistic functional group. Additionally, functional group adducts may not form stoichiometrically in all circumstances and the ideal strategy should take this into account. Therefore, strategy analysis can be performed in one of three modes: stoichiometrically where adduct formation can determine the precise number of functional groups, non-stoichiometrically where adduct formation can only determine whether a group is present and pseudostoichiometrically where adduct formation can determine if there is one or two instances of a functional group precisely but it cannot distinguish among 3 or more instances.

Furthermore, the number of instances of each functional group can be determined in a number of manners as we detect overlapping and subgraphs of each functional group. The strategy analysis was ran considering distinct functional groups only, distinct + overlapping, distinct + subgraph, distinct + subgraph + overlapping, distinct + subgraph + overlapping + super, and super functional groups only. Distinct only represents the functional groups likely to be detected by the most specific of adduct forming compounds, while other permutations allow us to consider the detection of functional groups in more permissive contexts. The increase in percent distinguishable compounds using the strategies generated by our analysis can guide researchers in both using commercially available adducts and guide development of new adducts.

### Computational platforms and libraries

All timed analyses were done on three identical machines with dual Xeon X5650 processors @ 2.67 GHz and 24 GB of 1333 MHz ECC memory running Fedora 18 “Spherical Cow.” All three algorithms were implemented in Perl 5.16.3 and SQLite v3.7.13 with DBI 1.631 was used in all programs interacting with a SQLite database.

## Results

### Algorithm performance

**Figure 8:**
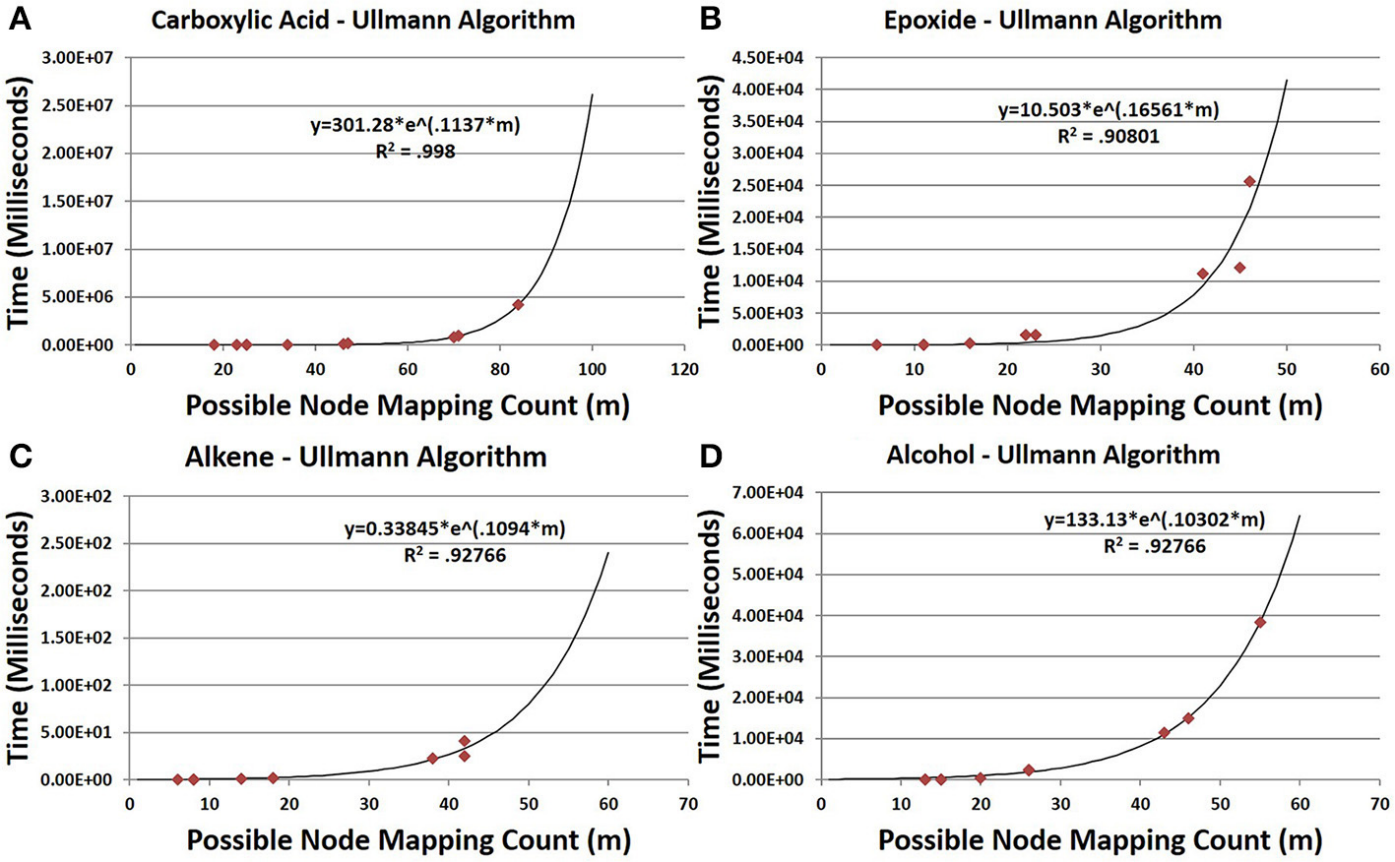
**Time trials for the Ullmann algorithm**. In all cases, the time needed for the Ullmann algorithm to find all instances increases essentially exponentially with increasing *m*. Data were analyzed by non-linear regression to *t* = aexp(bt). **(A,B,D)** The time to search for carboxylic acids, epoxides and alcohols shows exponential growth with respect to *m*. **(C)** The time needed to search for alkenes, while also exponential, is much smaller than that needed for all other functional groups, as the alkene group is the smallest of the four groups in both number of bonds and atoms. This indicates that the time needed to find a specific group varies with respect to its size in a strong nonlinear exponential manner (alcohol with only one more bond and atom takes much longer for *m* > 25).

**Figure 9:**
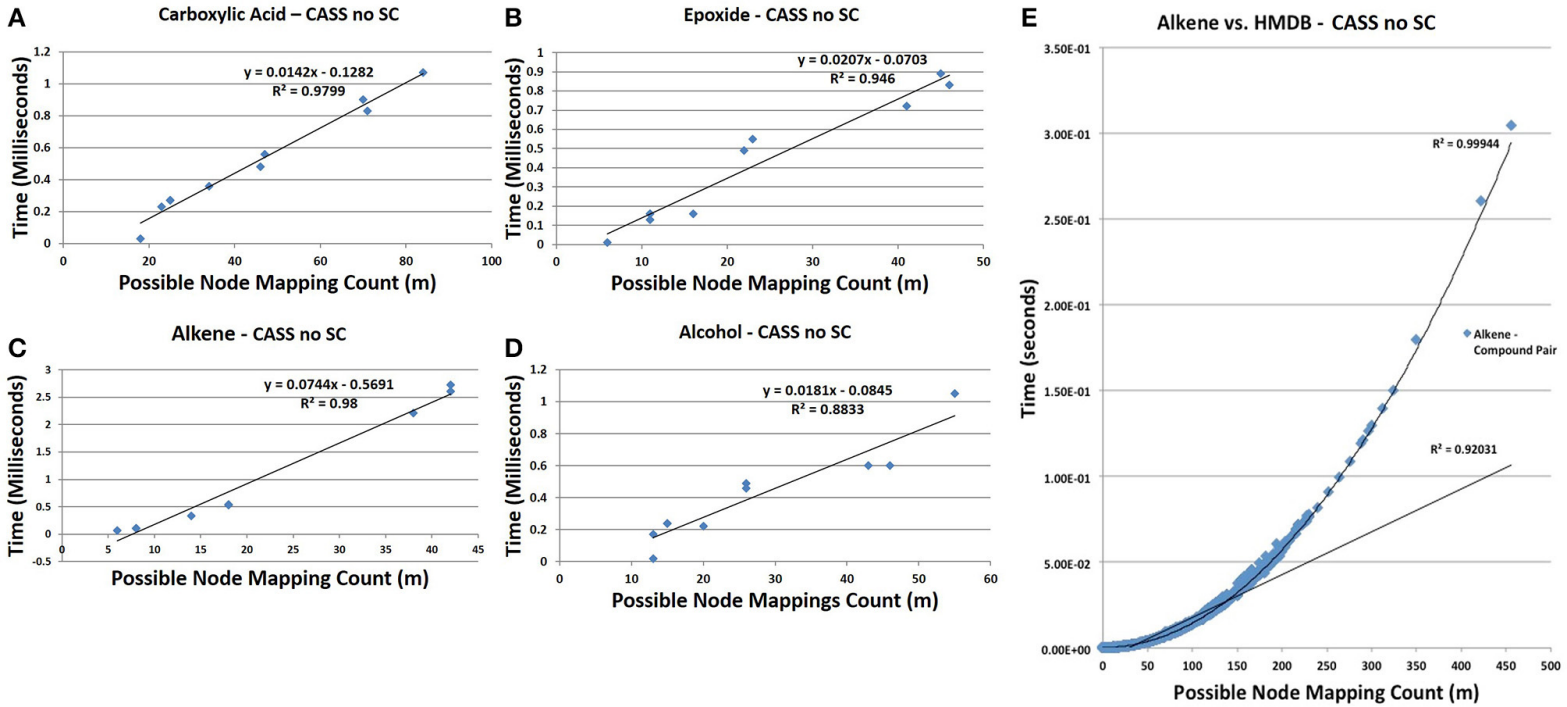
**Time trials for CASS with no short circuiting. (A)** The time needed to find carboxylic acids is pseudo-linear at *m* < 100 (*R*^2^ = 0.9799). **(B)** Similar to carboxylic acids, the time needed to find epoxides is pseudo-linear for observed *m*, (*R*^2^ = 0.946). **(C)** Similarly, the time needed to search for alkenes grows pseudo-linearly with *m* (*R*^2^ = 0.98). **(D)** The time needed to find alcohols remains low at all observed values of *m* but is less strongly linear than with other functional groups (*R*^2^ = 0.8833). It is likely that with respect to high values of *m*, all functional groups would show polynomial growth; however, for most values compounds, *m* will be sufficiently small to allow our algorithm to show pseudo-linear performance. **(E)** The time needed to find all alkenes in the HMDB demonstrates the non-linear performance of CASS for values of *m* > 150, as the overall trend matches a second-order polynomial with an *R*^2^ of 0.9994. Although non-linear, the time needed grows slowly enough to allow all functional group searches to complete in a relatively short amount of time. To estimate an upper bound on the values of *m* likely to be observed during functional group searching within metabolic databases, the value of *m* for each pair of functional group with database compound within the HMDB was determined. The values of *m* for each functional group were recorded and the largest value of *m* for each functional group was selected to create a set of the largest observed values of *m*. This set of largest values of *m* represents the most strenuous calculations that must be performed by our algorithm. The average largest value of *m* is 1007 with a standard deviation of 508. The largest value of *m* observed for all pairs was 4104. Although, these largest values of *m* are within the non-linear performance region of CASS, these values of *m* are still small enough to allow for efficient functional group searching within any given database structure. The time needed to find the other three functional groups in all HMDB entries was also determined and shown in Figure S2.

**Figure 10:**
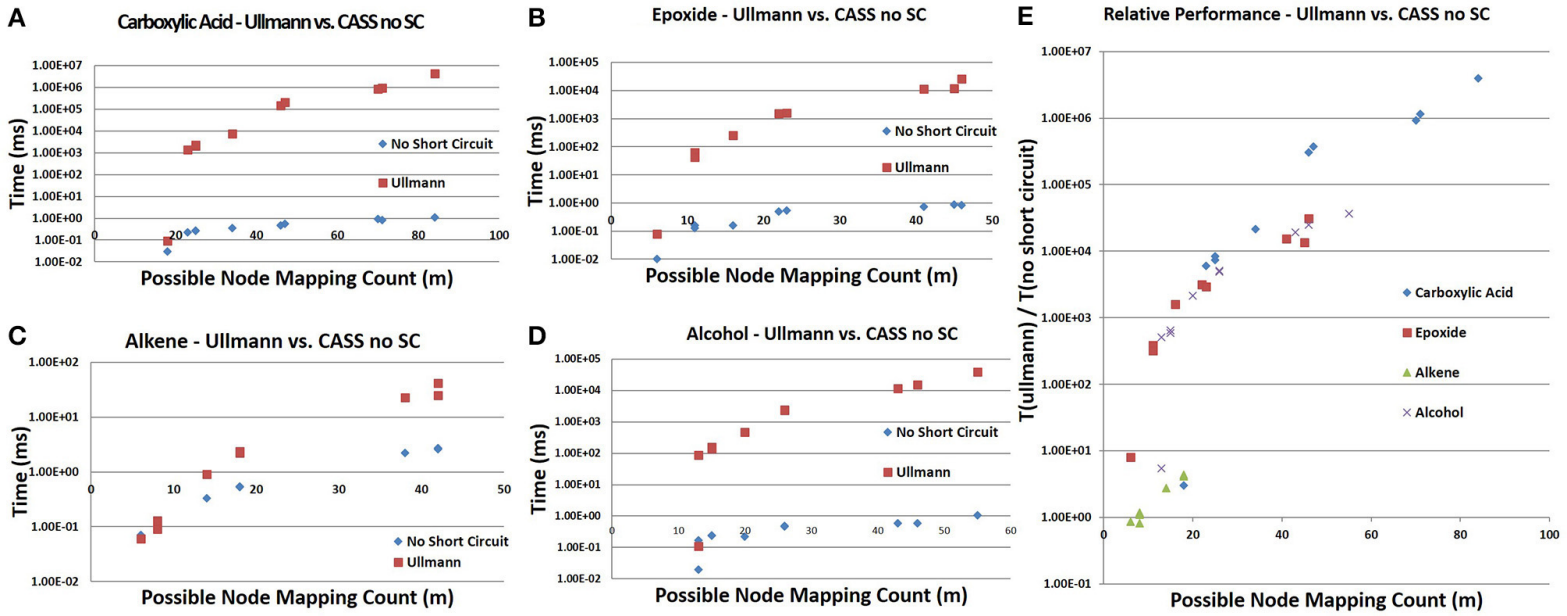
**Direct comparison of the Ullman algorithm to CASS with no short circuiting. (A–D)** For all four functional groups, our algorithm shows linear performance at these values of *m* while the Ullmann algorithm does not. **(E)** The ratio of the time needed by Ullmann vs. our algorithm with respect to *m* demonstrates that our algorithm is faster than the Ullmann algorithm in all cases. This ratio increases with *m* and varies between functional groups.

CASS outperforms the older Ullmann algorithm significantly when searching for functional groups within molfile files. The older Ullmann algorithm takes a prohibitively long amount of time for all but the most trivial analyses, while our algorithm readily performs in applications utilizing large numbers of molfile files. The number of atoms for a set of representative database compounds and functional groups was determined as was the possible node mapping count (m) for each functional-group/database-compound pair (Tables 1, 2). The relationship between m and algorithm performance becomes apparent in Figures 8–10. Figures 8, 9, based on Tables S1, S2 in Supplementary Material, visualize the obvious differences in performance between the Ullmann algorithm and CASS with no short-circuiting, in identifying four common functional groups in ten molfile files. The non-linear behavior of the Ullmann algorithm as shown in Figure 8 is clearly unsuitable for our functional group searching. The pseudo-linear behavior of our new algorithm as shown in Figure 9 is stable for values of m up to 150 and remains sufficiently fast for large values of m during functional group searching, making CASS tractable for systematic functional group searches in KEGG and HMDB. Furthermore, the demonstrated polynomial behavior of our algorithm (Figure 9E) is the best expected performance, given the debate on whether the common subgraph isomorphism problem has polynomial or NP-complete behavior (de Melo et al., 2013). Also, Figure 10 further highlights the relative differences between the Ullmann algorithm and CASS on a log scale. This difference in performance increases substantially with respect to *m*.

**Table 1:** **List of representative database compounds and functional groups**.

**Atoms and bonds in database compounds and functional groups**			
	**Compound name**	**Atoms**	**Bonds**
Database compounds	Deoxycytidine	29	30
	R-3-Hydroxybutyric acid	15	14
	2-Hydroxybutyric acid	15	14
	Deoxyuridine	28	29
	1-Methylhistidine	23	23
	Cortexolone	55	58
	2-Methoxyestrone	46	49
	Deoxycorticosterone	43	69
	1,3-Diaminopropane	15	14
	2-Ketobutyric acid	13	12
Functional groups	Carboxylic acid	5	4
	Epoxide	3	3
	Alkene	6	5
	Alcohol	3	2

The number of atoms and bonds in the database compound and in the functional group being searched for have an indirect impact on the performance of each algorithm.

**Table 2:** **Possible node mapping counts for paired functional group/database compound searches**.

**Possible node mapping count (*m*)**				
**Database compounds**	**Functional group**			
	**CA**	**Epoxide**	**Alkene**	**Alcohol**
Deoxycytidine	46	22	18	26
R-3-Hydroxybutyric acid	25	11	8	15
2-Hydroxybutyric acid	25	11	8	15
Deoxyuridine	47	23	18	26
1-Methylhistidine	34	16	14	20
Cortexolone	84	46	42	155
2-Methoxyestrone	71	41	38	46
Deoxycorticosterone	70	45	42	43
1,3-Diaminopropane	18	6	6	13
2-Ketobutyric acid	23	11	8	13

The possible node mapping counts (m) between functional group atoms and database compounds have a direct impact on algorithm performance. In most cases, larger functional groups have larger m values than smaller functional groups and therefore should take longer to search for. These values of m are based on an element node coloring scheme.

However, the improvement in our new algorithm with short-circuiting is sporadic (Figure 7B and Table S3) and is dependent on the order of the search of **M** and the number of valid isomorphic mappings in **M** (i.e., number of isomorphic **M'**). But an excellent case for utilizing the short-circuiting variant of our algorithm is when searching for stereoisomeric compounds within databases. Two large stereoisomeric compounds, A and B, will have a very large number of possible mappings as they contain an identical number and type of atoms. A single valid mapping of all atoms in A to all atoms in B is sufficient to determine that A and B are stereoisomeric. Additional valid mappings beyond the first convey no additional information regarding the relationship of compounds A and B and do not need to be determined. For a number of possible stereoisomers from KEGG Ligand, both the short-circuiting and non-short circuiting algorithms were compared, providing sporadic results where the short-circuiting either performed better or comparably to the non-short circuiting algorithm (Table S4 in Supplementary Material).

### Systematic isomer analysis

**Figure 11:**
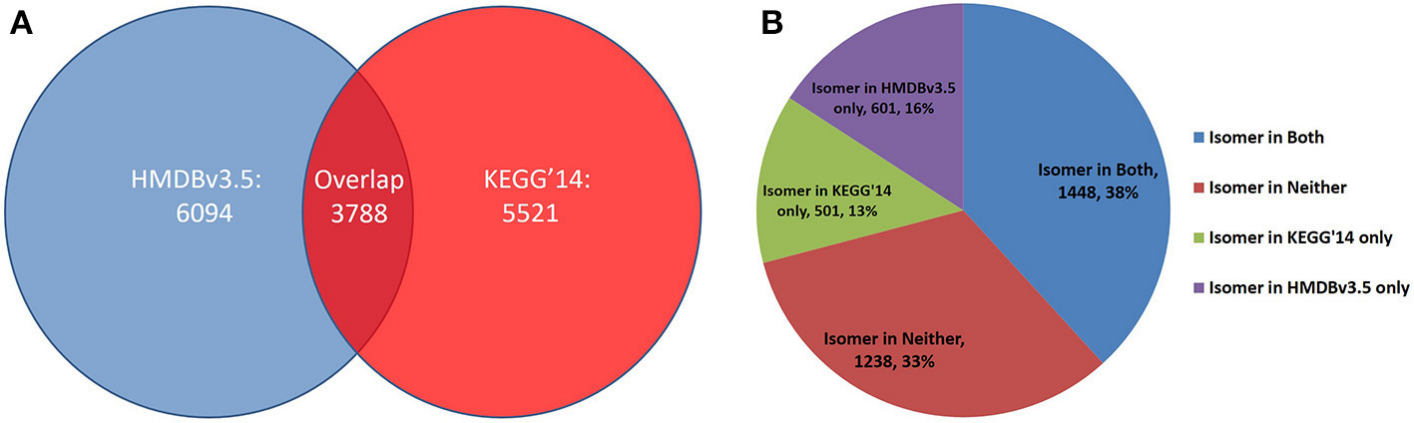
**Distinct molecular Formulae distributions across HMDBv3.5 and KEGG Ligand. (A)** In each database, the number of distinct formulae was determined. Each formula was tested to see if it existed in the other database. Of a total 15,403 formulae, only 3788 (24.6%) exist in both. **(B)** Each shared formula was compared against the HMDBv3.5 and KEGG'14 to test for isomerism in either database. The majority are isomers in either both or neither database, but some are isomers in one database exclusively.

The increased performance of CASS compared to the Ullmann algorithm allows for the rapid detection of functional groups and stereoisomers, which we used to create functional group-resolved SQLite versions of metabolite databases. From our functional group-resolved SQLite versions of the HMDBv3.5 and KEGG Ligand (as of March 2014), several additional analyses were performed. First, the number of distinct molecular formulae in both databases was determined as well as the number of molecular formulae the two databases have in common (Figure 11A). The 3557 molecular formulae were then compared against both databases to determine if the molecular formula was isomeric in neither, both, or either database. 39% were isomers in neither database, 32% were isomers in both, while 17 and 12% were isomers only in the HMDB and KEGG, respectively (Figure 11B).

**Figure 12:**
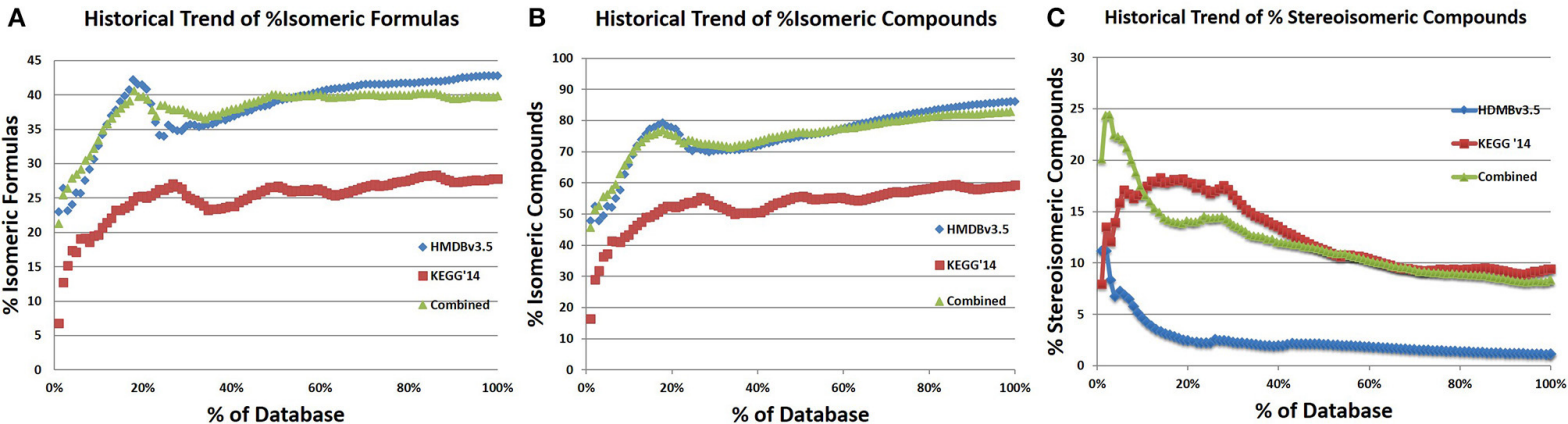
**Historic trends of % isomeric molecular formulae and compound entries. (A)** The historical trend in % isomeric formulae was determined by analyzing the isomeric content ofa growing percentage of the database, following the sorted order of IDs. This was done by iteratively adding 1% of each database to a temporary database, calculating how many distinct formulae there are in the database and the number of formulae for which there is only one compound, which is the number of isomeric formulae. For both HMDBv3.5 and KEGG'14, the % isomeric formulae increase with the number of compounds, but both seem to plateau. **(C)** The combined analysis was performed identically to the individual analysis, but 1% of both databases sorted by ID was added after each trial. The combined analysis reveals that the % isomeric formulae seems to plateau at around 40%, remaining at that level from 55% onwards. **(B)** In contrast, the % of isomeric compounds in the databases seems to grow at a slow but constant rate for the HMDB, (fits linear with *R*^2^ of 0.9883 after 30%) and a fluctuating but slowly increasing rate for KEGG.

In addition to determining the shared isomers between the databases, a historic trend analysis of isomerism was performed (Figures 12A–C). The percentage of isomeric molecular formulae in the HMDV3.5 and a combined database appear to have plateaued at 43 and 46%, respectively (Figures 12A,C). KEGG has reached a 28% isomeric content based on molecular formulae. This lower percentage of isomers in KEGG is likely due to the inclusion of pharmaceuticals and synthetic compounds that have unique molecular formulae that are not found in nature, which is probably why the isomeric content has not plateaued. What is also interesting is that the percent isomeric entries in all three databases (Figure 12B) is appreciably higher than the percent isomeric molecular formulae, indicating that a moderate number of isomeric molecular formulae are represented by more than 2 isomeric entries.

### Systematic stereoisomer analysis

Additionally, the higher performance of CASS allows for the comparison of two database structures in order to determine if they are stereoisomers of one another. For each database, all compound pairs in which the two compounds have identical formulae and the same number of bonds are tested for potential stereoisomerism. Since a single isomorphic instance of one database entry in another is sufficient to identify stereoisomeric compounds, our short-circuiting can be used to greatly accelerate these comparisons. As database structures can be very large, the potential number of mappings must be kept small for efficient analysis; this is achieved using 2-bond and 3-bond node coloring.

In addition to searching for stereoisomerism within each database, stereoisomerism was checked for compounds with the same formula and number of bonds between the two databases. Entries with duplicate names were excluded from this analysis to reduce the likelihood of comparing identical entries. The percentage of stereoisomeric compounds in the HMDBv3.5 and KEGG is 1.14 and 9.43%, respectively. The combined database has a percent stereoisomerism of 8.3%. Additionally, a historical trend of stereoisomers in HMDBv3.5, KEGG, and the combined database show early instability, followed by a downward trend that is plateauing. The large difference in stereoisomerism between KEGG and HMDB likely reflects the different portions of metabolism best represented by either database. The HMDB contains a large number of lipids and large aliphatic structures that typically have numerous structural isomers but few stereoisomers while KEGG has numerous sugars and other structures with a high number of potential stereoisomers.

### CS-tagging strategy analysis

All instances of each functional group were identified in a combined KEGG and HMDB database with duplicate entries removed. Using the functional group-resolved SQLite version of the combined KEGG and HMDB database with duplicate entries removed, we systematically tested different experimental CS-tagging strategies to determine, optimal strategies with 3, 5, 10, or 15 functional group adducts (Tables S5A–C in Supplementary Materials).

**Figure 13:**
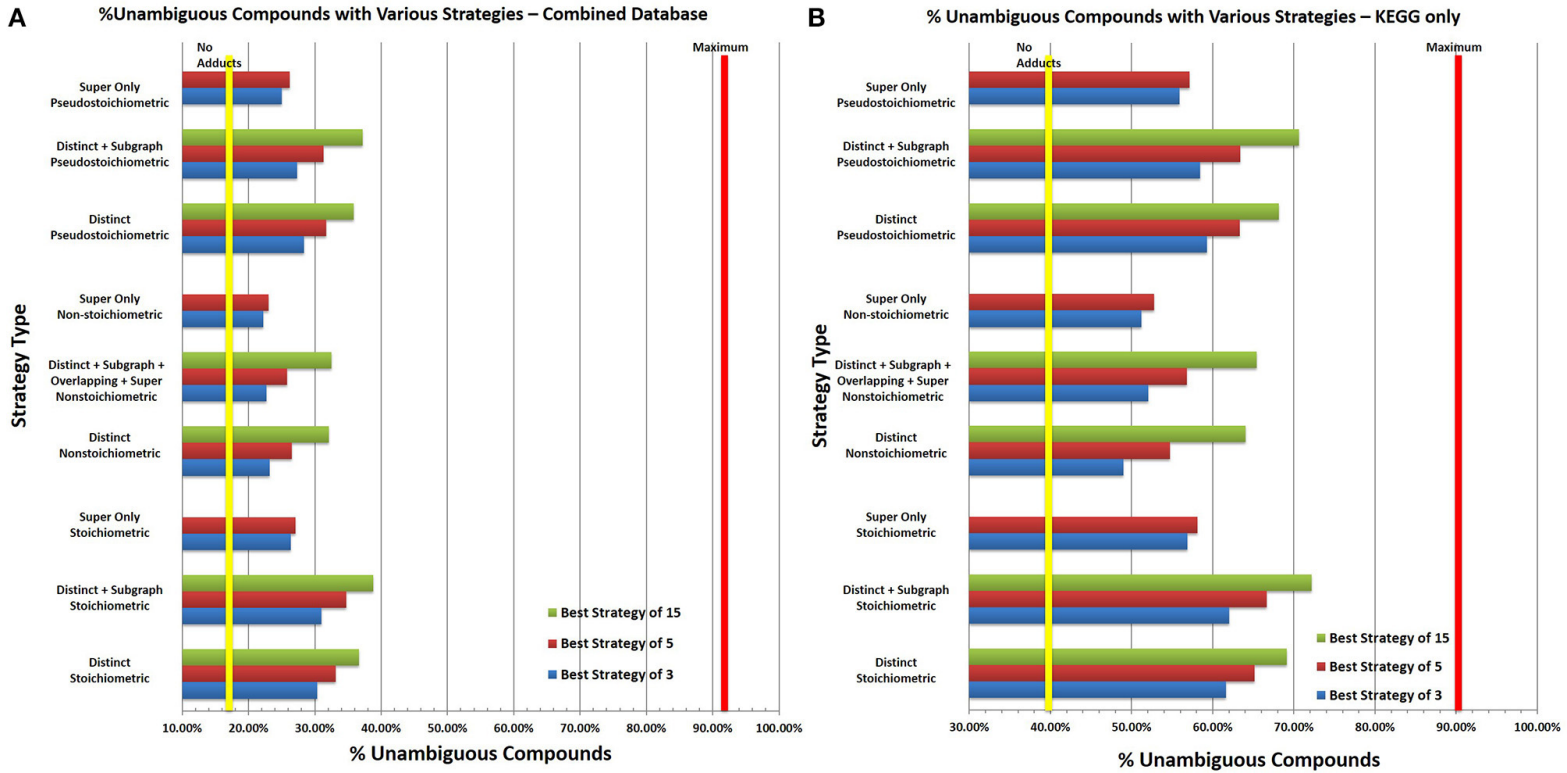
**Percent of Unambigiuous Compounds with Selected CS-Tagging Strategies. (A)** In general, using more adducts improves the disambiguation of compounds for all strategies. For pseudostoichiometric and stoichiometric strategies, the difference seen in utilizing 15 functional groups compared to 5 is roughly equal to the difference in performance seen with 5 and 3 functional groups indicating that multiplexing multiple groups offers increased performance but with diminishing returns. The yellow vertical line represents the percent of unambiguous formulae without adduct formation, the red line represents the percent of formulae without stereoisomers, which cannot be disambiguated with CS-Tagging alone due to stereoisomerism. In all three stoichiometric environments, very good performance is achieved by detecting distinct instances of the functional groups alone. The ability to detect subgraph or overlapping instances of each functional group offers only marginal improvements in performance. Super functional group only strategies consistently provided the worst performance. Stoichiometric tagging offers the maximum percent of disambiguation; however, it will be difficult to ensure consistent results experimentally. Pseudostoichiometric adduct formation provides performance close to stoichiometric tagging and much greater performance than non-stoichiometric tagging. However, the performance of all the strategies falls short of the theoretical maximum expected from stereoisomerism alone. This is likely the result of the distribution of isomers in the HMDB. Many structural isomers in the HMDB differ only slightly and map to a relatively small number of formulae, making them difficult to disambiguate. The functional groups comprising each strategy differ slightly; please refer to Tables S5A–C to find the set of functional groups comprising each strategy shown as well as the performance of additional strategies. **(B)** Similarly the same analysis was performed using the KEGG database only. As with 13A, the yellow line represents the percent of unambiguous compounds without adduct formation and the red line the theoretical maximum based on stereoisomerism within the database. As with the combined database, stoichiometric strategies outperform non-stoichiometric and pseudostoichiomeric strategies but pseudostoichiometric strategies have only slightly lower performance. The highest performing strategies allow for coverage of over 70% of all database compounds but stills fall short of the theoretical 90.6% maximum (see Tables S6A–C for the functional groups comprising each strategy). The better performance of the various strategies in KEGG compared to the combined database is the result of the different distribution of isomeric compounds in KEGG. Unlike the HMDB, the isomeric compounds in KEGG are more evenly distributed among KEGG molecular formulae, allowing easier disambiguation of isomeric compounds (see Figure S1 in Supplementary Material).

In all tests, 15 iterations were performed, with the top 50 strategies kept in the first iteration and the top 15 strategies kept in subsequent iterations, and, with a performance cutoff of 0.1%. The analysis was repeated under stoichiometric, non-stoichiometric and pseudostoichiometric quantification expectations and different degrees of allowed overlap between functional groups. Selected strategy types were compared against no adduct formation to visualize the improvements in compound disambiguation (Figure 13). Unfortunately the distribution of isomers within the HMDB makes it a poor representation of the effectiveness of CS-tagging strategies. Over 53% of the isomeric compounds in the HMDB are isomers of 9 or more other compounds (Figure S1). This level of high isomerism within the HMDB is due to the inclusion of a very large number of lipids and triglycerides, many of which are structural isomers of one another (different positions of double bonds in the acyl chains and positions of acyl chains on the backbone) and cannot be easily disambiguated by CS-tagging and MS alone. Additional information from other methods such as LC and tandem MS will be needed to resolve lipid structural isomerism, especially for triglycerides. KEGG on the other hand has a much more manageable isomer distribution. Strategy analysis was performed using both the combined HMDB and KEGG database as well as KEGG separately.

Stoichiometric adduct formation consistently generates the best increases in percent unambiguous compounds for both databases and the ideal strategy of 3 functional groups varies very little with varying the amount of overlap or with which database was analyzed. The optimal three adducts with distinct functional groups only increases the percent of unambiguous compounds in the combined database and the KEGG database from 17.13 to 30.35% and from 40.98 to 61.63%, respectively. Strategies with 15 functional groups perform slightly better with performances of 36.67% for the combined database and 69.13% for KEGG alone. Allowing for detection of overlapping, subgraph or super functional groups offers only minimal improvement; less than 1% for 3 functional groups and less than 2.5% for strategies of 15 functional groups.

In contrast, non-stoichiometric strategies provide the worst increases in percent unambiguous compounds. For the combined database, the ideal 3 functional group strategy only allows for 23.18% of compounds to be uniquely identified. The performance is better in KEGG alone, with the ideal strategy of 3 allowing 49% of compounds to be uniquely identified. As with stoichiometric analysis, ideal strategies are similar between the combined and KEGG database, however, in non-stoichiometric analysis, allowing for detection of overlapping, subgraph or super groups does allow for noticeable improvements for smaller strategies. Detection of overlapping, subgraph or super groups has an unpredictable effect on the performance of each strategy depending on what database is considered and the number of functional groups. In the combined database, detection of overlapping, subgraph or super groups decreases performance of three functional group strategies by a marginal amount, while for KEGG, marginal improvements are observed. However, their detection improves performance of all strategies with 10 or more functional groups in both databases marginally.

In reality due to the complexity and differing reactivity of metabolites, stoichiometric adduct formation is unlikely to occur for all compounds. However, pure non-stoichiometric adduct formation is unlikely to occur as well; adduct formation will likely occur in a pseudostoichiometric manner, wherein only one to three instances of a functional group can be reliably identified in a stoichiometric manner. Pseudostoichiometric strategies perform significantly better in both databases than non-stoichiometric strategies but only marginally worse than stoichiometric ones. For the combined and KEGG databases, the best pseudostoichiometric strategy of 3 allows for unique identification of 28.37 and 59.32% of compounds. The performance of these strategies increases steadily up to 15 functional groups for both databases up to 35.83 and 68.13% for the combined and KEGG databases, respectively. Detection of overlapping, subgraph, and super functional groups has a mixed effect for strategies with less than three functional groups, but is marginally helpful for all strategies with greater than 5 functional groups.

Additionally, strategies were generated using only the super functional groups under stoichiometric, pseudostoichiometric, and non-stoichiometric conditions. In all cases, the super only strategies delivered the worst performance by a significant margin and the algorithm terminated early due to the performance cutoff in all cases.

Collectively the optimal strategies determined by this analysis can be generalized to help aid in CS-tagging reagent development and use. The most common functional groups in strategies with five or fewer functional groups are alkene, methyl, ketone, carboxylic acid, dialkyl ether, and enol; therefore, adducts for these functional groups will allow for the greatest disambiguation of metabolites. Although reagents already exist for forming adducts with most of these groups, no CS-tagging agent exists for methyl groups nor can one be easily developed due to the group's lack of chemical reactivity. However, supplementary techniques such as NMR could be used in lieu of a CS-tagging agent to determine the number of methyl groups pseudostoichiometrically. Additionally, the marginal performance increases achieved by allowing the detection of overlapping, subgraph and super functional groups in addition to distinct instances of each functional group, indicates that reagents that can detect instances of functional groups within other chemical moieties will not be necessary for effective CS-tagging strategies. Instead, multiple reagents capable of forming adducts pseudostoichiometrically or stoichiometrically against specific moieties should be multiplexed. The poor performance of the super only strategies demonstrate that optimally, reagents should form adducts with functional groups that are neither exceedingly rare within the database nor ubiquitous.

## Discussion

Our new algorithm, CASS, significantly outperforms the Ullmann algorithm in finding complete isomorphisms in chemical structures. Although the prototypical solution to the MCSI problem and by extension the common subgraph isomorphism problem that we have solved, the modernization of the Ullmann algorithm shows that it not suitable for identifying identical regions between compounds. Additionally, the modernization of the Ullmann algorithm revealed a typographical mistake in the original publication.

CASS allows for the creation of functional group-resolved databases necessary for assigning functional group resolved molecular formulae derived from FT-MS analysis of CS-tagged metabolites to specific chemical structures. Additionally, the short-circuiting and advanced node coloring abilities of CASS allows the detection of all stereoisomers in the KEGG and HMDB metabolite databases within a few hours on a single midrange workstation (less $5K). We use CASS to determine the theoretical number of compounds (~9%) that cannot be distinguished using the combined functional group (from CS-tagging) and molecular formula (from FT-MS) information.

Furthermore, conversion of the molfile flat file databases into SQLite provides a number of advantages such as portability, ease of query with CS-tagging and molecular formula data as well as improvements in database access speed. Also, our variant of the molfile file format expands on the traditional file format, enabling the designation of more complex substructures within specific chemical contexts. This is achieved by allowing dynamic element typing for given atoms and support for contextual atoms to delineate functional groups with common features (e.g., aldehydes and ketones). Additionally, unlike many previous functional group search programs, CASS does not require hard coding in order to search for a given structure; therefore, the end user can easily add, remove, or modify functional groups to his or her choice without introducing errors into the program.

Our analysis of the HMDBv3.5 and KEGG Compound'13 shows only a low amount of overlap as only 24% of the distinct formulae from each databases exist in both. Thus, current database searches for metabolites based on molecular formulae could be biased, depending on the choice of the database. In addition, the significant presence of isomeric molecular formulae in these databases (i.e., 43% in HMDBv3.5, 28% in KEGG Compound 13', and 46% in a combined database) indicates that additional structural features such as functional groups determined by CS-tagging will need to be included in molecular formula-based database searches to facilitate unambiguous metabolite assignment of a large number of detected mass peaks. Moreover, a unique assignment of a molecular formula in one database could map to multiple compounds in another. Therefore, unique assignments should be checked in multiple databases to prevent potential misidentification of MS-detected compounds.

As an aside, the apparent plateauing at roughly 46% percent isomeric compounds in a combined database (from HMDBv3.5 and KEGG'13) may indicate a biologically relevant percent isomeric content of metabolomes in the biosphere. This would naturally be due to the significant number of stereospecific enzyme-catalyzed chemical reactions in cellular metabolism that appears to maintain an approximately 50% stereospecific chemical environment in living systems. The specific biological significance of this phenomenon is not completely apparent, but we suspect it may be due to some fundamental principle in information theory that living systems take advantage of at the stereochemical level.

Also, our analysis of CS-tagging strategies indicate that by multiplexing several functional group derivatizations in a single sample, using the unique isotope labeling distributions inherent in the design of the reagents, it is possible to determine: (i) the numbers of distinguishable metabolites having each functional group, (ii) the exact mass of the desired radical with high resolution MS, and (iii) chemical shift and molecular connectivity information with NMR. Together these can distinguish between many isomeric species with the same molecular formula but different functional groups, and therefore greatly reduce the ambiguity of structural assignment, especially for non-lipid metabolites. However, isomeric disambiguation of lipids will require additional methods that identify specific substructure.

In conclusion, by coupling molecular formula determination from ultra-high resolution FT-MS with additional chemical substructure information like functional group identification from CS-tagging or substructure determination from tandem MS-MS or NMR, our chemically aware substructure search algorithm CASS can provide robust assignment of FT-MS raw data to various metabolites and their isotopic enrichment profiles (e.g., ^13^C isotopologs of UDP N-acetylglucosamine or UDP-GlcNAc) in SIRM studies. The identity and fractional enrichment of labeled metabolites thus obtained are valuable parameters for modeling the contribution of various pathways to the synthesis of given labeled metabolites from tracer precursors such as done for UDP-GlcNAc synthesis from ^13^C_6_-glucose (Moseley et al., 2011). Thus, the combined molecular formula and chemical substructure-based computational tools described here are key components of our computational pipeline to facilitate systems biochemical understanding of human metabolome and its perturbations by disease development.

### Conflict of interest statement

The authors declare that the research was conducted in the absence of any commercial or financial relationships that could be construed as a potential conflict of interest.
