# Supplementary material for: Development and in silico evaluation of large-scale metabolite identification methods using functional group detection for metabolomics
Source: Front Genet. 2014 Jul 28;5:237. doi: 10.3389/fgene.2014.00237 (PMC4112935; doi:10.3389/fgene.2014.00237)
Supplement: Supplementary file 1 [file Presentation1.PDF]

Supplementary Materials:

Table S1: Performance of test functional group searches using the Ullmann algorithm. Although the Ullmann algorithm finds all subgraphs for a given compound and functional group pairing, the time needed is prohibitive for functional group searches against a large number of database compounds or for large compounds and large functional groups. For example, searching for the carboxylic acid functional group within reasonably large structures such as deoxycorticosterone takes over one hour.

Table S1: Search time trials using Ullmann algorithm

		Functional Group			
		Carboxylic Acid	Epoxide	Alkene	Alcohol
Database Compounds	Deoxycytidine	145828ms	1548ms	2.36ms	2422ms
	R-3-Hydroxybutyric Acid	1992ms	62ms	.13ms	144ms
	2-Hydroxybutyric Acid	2275ms	62ms	.12ms	142ms
	Deoxyuridine	210620ms	1608ms	2.21ms	2340ms
	1-Methylhistidine	7651ms	256ms	.91ms	473ms
	Cortexolone	4239178ms	25635ms	41.41ms	38455ms
	2-Methoxyestrone	955437ms	11167ms	22.97ms	15019ms
	Deoxycorticosterone	828605ms	12080ms	24.81ms	11490ms
	1,3-Diaminopropane	.09ms	.08ms	.06ms	.11ms
	2-Ketobutyric Acid	1375ms	42ms	.09ms	86ms

Table S2: Performance of test functional group searches using CASS with no Short Circuiting. Our algorithm finds all functional groups considerably faster than the original Ullmann algorithm. The

time needed to find a particular functional group increases with the number of atoms in both the functional group and database compound, but the time needed for a search is most related to the number of possible atom to atom mappings between the functional groups and database compounds. Small functional groups show nearly no increase in time as the number of possible mappings does not increase very quickly. However, this pseudo-linear performance only occurs for values of m lower than approximately 150 but remains sufficiently fast to allow for efficient functional group searching in all database compounds (Figure 9E).

Table S2: Search time trials using CASS with no Short Circuiting

		Functional Group			
		Carboxylic Acid	Epoxide	Alkene	Alcohol
Database Compounds	Deoxycytidine	.48ms	.49ms	.54ms	.49ms
	R-3-Hydroxybutyric Acid	.27ms	.16ms	.11ms	.24ms
	2-Hydroxybutyric Acid	.27ms	.16ms	.11ms	.24ms
	Deoxyuridine	.56ms	.55ms	.53ms	.46ms
	1-Methylhistidine	.36ms	.16ms	.33ms	.22ms
	Cortexolone	1.07ms	.83ms	2.72ms	1.05ms
	2-Methoxyestrone	.83ms	.72ms	2.21ms	.60ms
	Deoxycorticosterone	.90ms	.89ms	2.61ms	.60ms
	1,3-Diaminopropane	.03ms	.01ms	.07ms	.02ms
	2-Ketobutyric Acid	.23ms	.13ms	.11ms	.17ms

Table S3: Performance of test functional group searches using CASS with Short Circuiting. This algorithm terminates when the first proper mapping is found. This allows for significant relative and absolute time savings for some database functional group pairings compared to our algorithm with short-circuiting disabled. For large database compounds and/or large functional groups, this time savings can be significant as is the case for 2-Methoxyesterone and Alkene. The time savings

of this algorithm depends on the amount of time needed to find the first valid mapping relative to the time needed to complete the enumeration procedure. The earlier the first valid mapping was found in the enumeration process the greater the time savings. Therefore, short circuiting is most effective when the algorithm happens to find a valid mapping early in the enumeration process or when there are numerous instances of the functional group.

Table S3: Search time trials using CASS with Short Circuiting

		Functional Group			
		Carboxylic Acid	Epoxide	Alkene	Alcohol
Database Compounds	Deoxycytidine	.48ms	.49ms	.23ms	.29ms
	R-3-Hydroxybutyric Acid	.19ms	.16ms	.11ms	.12ms
	2-Hydroxybutyric Acid	.19ms	.16ms	.12ms	.03ms
	Deoxyuridine	.55ms	.55ms	.22ms	.29ms
	1-Methylhistidine	.20ms	.16ms	.12ms	.06ms
	Cortexolone	1.07ms	.83ms	1.27ms	.28ms
	2-Methoxyestrone	.82ms	.73ms	.03ms	.06ms
	Deoxycorticosterone	.90ms	.89ms	2.6ms	.25ms
	1,3-Diaminopropane	.03ms	.01ms	.08ms	.02ms
	2-Ketobutyric Acid	.10ms	.13ms	.11ms	.10ms

Table S4: Performance comparison for stereoisomerism using CASS, with and without short circuiting. For non-stereoisomeric compounds, no valid mapping exists and therefore both algorithms must exhaust all possible enumerations before termination. As a result both algorithms performed identically when the two compound were non-stereoisomeric. However, when stereoisomerism was present, the short-circuiting algorithm terminates early saving considerable time. The extent of time savings from short-circuiting increases as the compounds become larger, allowing for the detection of stereoisomerism in a larger set of compounds than would be feasible with the non-short circuiting algorithm.

Table S4: Comparison of CASS with and without short circuiting for stereoisomerism testing.			
Number of atoms in compound pair	Short-circuit time (seconds)	Non-short circuit time (seconds)	Stereoisomer Detected Y/N
10	0.001	0.001	Y
10	0.001	0.001	N
23	0.003	0.01	Y
23	0.002	0.002	N
29	0.005	0.038	Y
29	0.001	0.001	N
46	0.01	0.077	Y
46	0.002	0.002	N
61	0.023	0.427	Y
61	0.02	0.02	N
66	0.005	0.004	N
75	0.135	0.134	N
88	0.966	0.966	N
93	0.015	0.014	N
158	0.102	0.101	N
158	0.613	0.616	N

Table S5: Functional groups comprising optimal strategies for the combined HMDB and KEGG database and their performance in percent unambiguous compounds. **A)** Stoichiometric analysis offers the best performance of all strategies and provides very good results with only three functional groups. However, stoichiometric adduct formation is likely impossible to ensure.

Table S5 A: Functional groups comprising best stoichiometric strategies for combined database				
**Strategy:**	**Best strategy of 3**	**Best strategy of 5**	**Best strategy of 10**	**Best strategy of 15**
**Distinct Functional Group Only**	Alkene, Methyl, Secondary Alcohol (30.35%)	Alkene, Ketone, Methyl, Primary Alcohol, Secondary Alcohol (33.12%)	1,2-diphenol, Alkene, Carboxylic Acid, Carboxylic Acid Ester, Ketone, Methyl, Phenol, Primary Alcohol, Secondary Alcohol, Tertiary Alcohol (35.7%)	1,2-diphenol, Aldehyde, Alkene, Carboxylic Acid, Carboxylic Acid Ester, Dialkylether, Imine, Ketone, Methyl, Phenol, Primary Amine, Primary Alcohol, Secondary Alcohol, Secondary Amine, Tertiary Alcohol (36.67%)
**Distinct Functional Group + Subgraph**	Alkene, Methyl, Secondary Alcohol (31.03%)	Alkene, Dialkylether, Enol, Methyl, Secondary Alcohol (34.78%)	Alkene, Dialkylether, Enol, Enolether, Ketone, Methyl, Phenol, Primary Alcohol, Secondary Alcohol, Secondary Amine (37.73%)	1,2-diol, 5-Heterocycle, Aldehyde, Alkene, Dialkylether, Enamine, Enol, Enolether, Ketone, Methyl, Phenol, Primary Amine, Primary Alcohol, Secondary Alcohol, Secondary Amine (38.77%)
**Distinct Functional Group + Overlapping**	Alkene, Methyl, Secondary Alcohol (31.06%)	Alkene, Ketone, Methyl, Phenol, Secondary Alcohol (33.93%)	6-Heterocycle, Alkene, Carboxylic Acid, Carboxylic Acid Ester, Ketone, Methyl, Phenol, Primary Alcohol, Secondary Alcohol, Secondary Amine (36.91%)	1,2-diol, 1,2-diphenol, 5-Heterocycle, 6-Heterocycle, Alkene, Carboxylic Acid, Carboxylic Acid Ester, Dialkylether, Ketone, Methyl, Phenol, Primary Amine, Primary Alcohol, Secondary Alcohol, Secondary Amine (38.06%)
**Distinct Functional Group + Overlapping + Subgraph**	Alkene, Methyl, Secondary Alcohol (30.62%)	Alkene, Dialkylether, Enol, Methyl, Secondary Alcohol (34.25%)	1,2-diol, 6-Heterocycle, Alkene, Dialkylether, Enol, Enolether, Ketone, Methyl, Secondary Alcohol, Secondary Amine (37.32%)	1,2-diol, 1,2-diphenol, 5-Heterocycle, 6-Heterocycle, Alkene, Alpha-aminoacid, Dialkylether, Enamine, Enol, Enolether, Ketone, Methyl, Primary Alcohol, Secondary Alcohol, Secondary Amine (38.37%)
**Distinct Functional Group + Overlapping + Subgraph + Super**	Alkene, Methyl, Secondary Alcohol (30.62%)	Alkene, Enol, Methyl, Secondary Alcohol, Super Ether (34.29%)	1,2-diol, 6-Heterocycle, Alkene, Enol, Enolether, Ketone, Methyl, Secondary Alcohol, Secondary Amine, Super Ether (37.37%)	1,2-diol, 1,2-diphenol, 6-Heterocycle, Alkene, Alpha-aminoacid, Enamine, Enol, Enolether, Ketone, Methyl, Primary Alcohol, Secondary Alcohol, Secondary Amine, Super Carboxylic Acid Derivative, Super Ether (38.48%)
**Super Only**	Super Carboxylic Acid Derivative, Super Ether, Super Hydroxyl (26.41%)	Super Amine, Super Carboxylic Acid Amide, Super Carboxylic Acid Derivative, Super Ether, Super Hydroxyl (27.08%)	Algorithm terminated due to performance cutoff	Algorithm terminated due to performance cutoff

Table S5 B) For non-stoichiometric strategy analysis, alcohols and amines have a greater impact earlier in the strategy analysis than in stoichiometric

strategies. Furthermore, functional groups such as methyl groups and alkenes impart much less performance in a non-stoichiometric environment than in a stoichiometric one.

Table S5 B: Functional groups comprising best non-stoichiometric strategies for combined database				
**Strategy:**	**Best strategy of 3**	**Best strategy of 5**	**Best strategy of 10**	**Best strategy of 15**
**Distinct Functional Group Only**	Alkene, Ketone, Secondary Alcohol (23.18%)	Alkene, Ketone, Methyl, Primary Alcohol, Secondary Alcohol (26.5%)	Alkene, Carboxylic Acid, Carboxylic Acid Ester, Dialkylether, Ketone, Methyl, Phenol, Primary Alcohol, Secondary Alcohol, Tertiary Alcohol (30.51%)	1,2-diol, 1,2-diphenol, Aldehyde, Alkene, Carboxylic Acid, Carboxylic Acid Ester, Dialkylether, Ketone, Methyl, Phenol, Primary Amine, Primary Alcohol, Secondary Alcohol, Secondary Amine, Tertiary Alcohol (32.1%)
**Distinct Functional Group + Subgraph**	Ketone, Methyl, Secondary Alcohol (22.94%)	Enol, Ketone, Methyl, Primary Alcohol, Secondary Alcohol (25.99%)	Carboxylic Acid Ester, Dialkylether, Enol, Enolether, Ketone, Methyl, Primary Alcohol, Secondary Alcohol, Secondary Amine, Tertiary Alcohol (30.51%)	1,2-diol, Aldehyde, Alkene, Carboxylic Acid Ester, Dialkylether, Enol, Enolether, Ketone, Methyl, Phenol, Primary Amine, Primary Alcohol, Secondary Alcohol, Secondary Amine, Tertiary Alcohol (32.66%)
**Distinct Functional Group + Overlapping**	Alkene, Ketone, Secondary Alcohol (23.29%)	Alkene, Ketone, Methyl, Primary Alcohol, Secondary Alcohol (26.3%)	5-Heterocycle, Alkene, Carboxylic Acid, Carboxylic Acid Ester, Ketone, Methyl, Phenol, Primary Alcohol, Secondary Alcohol, Tertiary Alcohol (30.86%)	5-Heterocycle, 6-Heterocycle, Aldehyde, Alkene, Alkylarylethermol, Carboxylic Acid, Carboxylic Acid Ester, Dialkylether, Ketone, Methyl, Phenol, Primary Alcohol, Secondary Alcohol, Secondary Amine, Tertiary Alcohol (32.89%)
**Distinct Functional Group + Overlapping + Subgraph**	Ketone, Methyl, Secondary Alcohol (22.61%)	Enol, Enolether, Ketone, Methyl, Secondary Alcohol (25.68%)	5-Heterocycle, Carboxylic Acid Ester, Enol, Enolether, Ketone, Methyl, Primary Alcohol, Secondary Alcohol, Secondary Amine, Tertiary Alcohol (30.53%)	1,2-diol, 5-Heterocycle, 6-Heterocycle, Aldehyde, Alkene, Carboxylic Acid Ester, Enamine, Enol, Enolether, Ketone, Methyl, Primary Alcohol, Secondary Alcohol, Secondary Amine, Tertiary Alcohol (32.45%)
**Distinct Functional Group + Overlapping + Subgraph + Super**	Ketone, Super Carboxylic Acid Derivative, Super Hydroxyl (22.7%)	Ketone, Methyl, Secondary Alcohol, Super Carboxylic Acid Derivative, Super Hydroxyl (25.78%)	5-Heterocycle, Enol, Enolether, Ketone, Methyl, Primary Alcohol, Secondary Alcohol, Super Carboxylic Acid Derivative, Super Ether, Tertiary Alcohol (30.54%)	1,2-diol, 5-Heterocycle, 6-Heterocycle, Aldehyde, Alkene, Enol, Enolether, Ketone, Methyl, Primary Alcohol, Secondary Alcohol, Secondary Amine, Super Carboxylic Acid Derivative, Super Ether, Tertiary Alcohol (32.55%)
**Super Only**	Super Carboxylic Acid Derivative, Super Ether, Super Hydroxyl (22.22%)	Super Amine, Super Carboxylic Acid Amide, Super Carboxylic Acid Derivative, Super Ether, Super Hydroxyl (23.06%)	Algorithm terminated due to performance cutoff	Algorithm terminated due to performance cutoff

Table S5 C) The pseudostoichiometric strategies for distinct functional groups are very similar to those that performed well in stoichiometric strategies.

When subgraphs and overlapping can be detected, pseduotstochiometric strategies differ from nonstoichiometric and stoichiometric strategies until more functional groups are added. Given the difficulty of ensuring stoichiometric adduct formation; these strategies will likely be most efficacious in the wet lab environment.

Table S5 C: Functional groups comprising best pseudostoichiometric strategies for combined database				
**Strategy:**	**Best strategy of 3**	**Best strategy of 5**	**Best strategy of 10**	**Best strategy of 15**
Distinct Functional Group Only	Alkene, Methyl, Secondary Alcohol (28.37%)	Alkene, Ketone, Methyl, Primary Alcohol, Secondary Alcohol (31.68%)	1,2-diphenol, Alkene, Carboxylic Acid, Carboxylic Acid Ester, Ketone, Methyl, Phenol, Primary Alcohol, Secondary Alcohol, Tertiary Alcohol (34.74%)	1,2-diphenol, Aldehyde, Alkene, Carboxylic Acid, Carboxylic Acid Ester, Dialkylether, Imine, Ketone, Methyl, Phenol, Primary Amine, Primary Alcohol, Secondary Alcohol, Secondary Amine, Tertiary Alcohol (35.83%)
Distinct Functional Group + Subgraph	Dialkylether, Methyl, Secondary Alcohol (27.35%)	Alkene, Dialkylether, Enol, Methyl, Secondary Alcohol (31.3%)	Alkene, Dialkylether, Enol, Enolether, Ketone, Methyl, Phenol, Primary Alcohol, Secondary Alcohol, Secondary Amine (35.71%)	1,2-diol, Aldehyde, Alkene, Carboxylic Acid Ester, Dialkylether, Enol, Enolether, Ketone, Methyl, Phenol, Primary Amine, Primary Alcohol, Secondary Alcohol, Secondary Amine, Tertiary Alcohol (37.18%)
Distinct Functional Group + Overlapping	Alkene, Methyl, Secondary Alcohol (28.7%)	Alkene, Ketone, Methyl, Phenol, Secondary Alcohol (32.1%)	5-Heterocycle, Alkene, Carboxylic Acid, Carboxylic Acid Ester, Ketone, Methyl, Phenol, Primary Alcohol, Secondary Alcohol, Secondary Ami (35.62%)	1,2-diphenol, 5-Heterocycle, 6-Heterocycle, Alkene, Carboxylic Acid, Carboxylic Acid Ester, Dialkylether, Ketone, Methyl, Phenol, Primary Amine, Primary Alcohol, Secondary Alcohol, Secondary Amine, Tertiary Alcohol (36.89%)
Distinct Functional Group + Overlapping + Subgraph	Dialkylether, Methyl, Secondary Alcohol (27.25%)	Alkene, Dialkylether, Enol, Methyl, Secondary Alcohol (31.08%)	5-Heterocycle, Alkene, Dialkylether, Enol, Enolether, Ketone, Methyl, Primary Alcohol, Secondary Alcohol, Secondary Amine (35.56%)	1,2-diol, 1,2-diphenol, 5-Heterocycle, 6-Heterocycle, Alkene, Alpha-aminoacid, Dialkylether, Enol, Enolether, Ketone, Methyl, Primary Alcohol, Secondary Alcohol, Secondary Amine, Tertiary Alcohol (36.89%)
Distinct Functional Group + Overlapping + Subgraph + Super	Ketone, Methyl, Super Hydroxyl (27.74%)	Enolether, Ketone, Methyl, Secondary Alcohol, Super Hydroxyl (31.29%)	5-Heterocycle, Alkene, Enol, Enolether, Ketone, Methyl, Primary Alcohol, Secondary Alcohol, Secondary Amine, Super Ether (35.61%)	1,2-diol, 1,2-diphenol, 6-Heterocycle, Alkene, Alpha-aminoacid, Enol, Enolether, Ketone, Methyl, Primary Alcohol, Secondary Alcohol, Secondary Amine, Super Carboxylic Acid Derivative, Super Ether, Tertiary Alcohol (37.03%)
Super Only	Super Carboxylic Acid Derivative, Super Ether, Super Hydroxyl (25.51%)	Super Amine, Super Carboxylic Acid Amide, Super Carboxylic Acid Derivative, Super Ether, Super Hydroxyl (26.18%)	Algorithm terminated due to performance cutoff	Algorithm terminated due to performance cutoff

Table S6 Functional groups comprising optimal strategies for the combined KEGG database and their performance in % unambigious formulas. **A)** As seen with the combined database, stoichiometric strategies allow for the best increases in percent unambigious formulas. Additionally the differences in the databases manifests itself most clearly in the increased diversity of functional groups comprising the best performing strategy of three groups.

Table S6 A: Functional groups comprising best stoichiometric strategies for KEGG only				
**Strategy:**	**Best strategy of 3**	**Best strategy of 5**	**Best strategy of 10**	**Best strategy of 15**
Distinct Functional Group Only	Alkene, Ketone, Methyl (61.63%)	Alkene, Carboxylic Acid, Ketone, Methyl, Secondary Alcohol (65.14%)	Aldehyde, Alkene, Carboxylic Acid, Ketone, Methyl, Phenol, Primary Alcohol, Secondary Alcohol, Secondary Amine, Tertiary Alcohol (68.15%)	1,2-diphenol, Aldehyde, Alkene, Carboxylic Acid, Carboxylic Acid Ester, Dialkylether, Imine, Ketone, Methyl, Phenol, Primary Amine, Primary Alcohol, Secondary Alcohol, Secondary Amine, Tertiary Alcohol (69.31%)
Distinct Functional Group + Subgraph	Alkene, Methyl, Secondary Alcohol (62%)	Alkene, Dialkylether, Enol, Methyl, Secondary Alcohol (66.65%)	Alkene, Dialkylether, Enol, Enolether, Ketone, Methyl, Phenol, Primary Alcohol, Secondary Alcohol, Secondary Amine (70.82%)	1,2-diol, Aldehyde, Alkene, Dialkylether, Enol, Enolether, Ketone, Methyl, Phenol, Primary Amine, Primary Alcohol, Secondary Alcohol, Secondary Amine, Tertiary Alcohol, Tertiary Amine (72.21%)
Distinct Functional Group + Overlapping	Alkene, Methyl, Secondary Alcohol (62.24%)	Alkene, Ketone, Methyl, Phenol, Secondary Alcohol (66.16%)	1,2-diol, 6-Heterocycle, Aldehyde, Alkene, Carboxylic Acid, Ketone, Methyl, Phenol, Secondary Alcohol, Secondary Amine (69.76%)	1,2-diol, 1,2-diphenol, 5-Heterocycle, 6-Heterocycle, Aldehyde, Alkene, Carboxylic Acid, Carboxylic Acid Secondary Amide, Dialkylether, Ketone, Methyl, Phenol, Primary Amine, Primary Alcohol, Secondary Alcohol (71.16%)
Distinct Functional Group + Overlapping + Subgraph	Alkene, Methyl, Secondary Alcohol (61.77%)	Alkene, Dialkylether, Enol, Methyl, Secondary Alcohol (66.5%)	1,2-diol, Alkene, Alpha-aminoacid, Dialkylether, Enol, Enolether, Ketone, Methyl, Secondary Alcohol, Secondary Amine (70.55%)	1,2-diol, 6-Heterocycle, Aldehyde, Alkene, Alpha-aminoacid, Dialkylether, Enamine, Enol, Enolether, Ketone, Methyl, Phenol, Primary Alcohol, Secondary Alcohol, Secondary Amine (71.91%)
Distinct Functional Group + Overlapping + Subgraph + Super	Methyl, Secondary Alcohol, Super Hydroxyl (61.94%)	Alkene, Enol, Methyl, Secondary Alcohol, Super Ether (66.69%)	1,2-diol, Alkene, Enol, Enolether, Ketone, Methyl, Secondary Alcohol, Secondary Amine, Super Carboxylic Acid Derivative, Super Hydroxyl (70.87%)	1,2-diol, 1,2-diphenol, 6-Heterocycle, Alkene, Alpha-aminoacid, Enamine, Enol, Enolether, Ketone, Methyl, Phenol, Secondary Alcohol, Secondary Amine, Super Carboxylic Acid Derivative, Super Hydroxyl (72.11%)
Super Only	Super Carboxylic Acid Derivative, Super Ether, Super Hydroxyl (56.9%)	Super Amine, Super Carboxylic Acid Amide, Super Carboxylic Acid Derivative, Super Ether, Super Hydroxyl (58.1%)	Algorithm terminated due to performance cutoff	Algorithm terminated due to performance cutoff

Table S6 B) The optimal non-stoichiometric strategies are similar to the stoichiometric strategies but allow for less disambiguation of database compounds. The performance difference between stoichiometric and non-stoichiometric strategies decreases as the number of functional groups within each strategy increases. Additionally, the ketone, secondary alcohol and dialkyl ethers perform relatively better non-stoichiometrically than stoichiometrically, providing better performance in some strategies than alkenes or methyl groups.

Table S6 B: Functional groups comprising best non-stoichiometric strategies for KEGG only				
**Strategy:**	**Top 3**	**Top 5**	**Top 10**	**Top 15**
Distinct Functional Group Only	Alkene, Ketone, Methyl (49%)	Alkene, Carboxylic Acid, Ketone, Methyl, Secondary Alcohol (54.76%)	Aldehyde, Alkene, Carboxylic Acid, Carboxylic Acid Ester, Ketone,Methyl, Phenol, Primary Alcohol, Secondary Alcohol, Tertiary Alcohol (61.54%)	1,2-diol, 1,2-diphenol, Aldehyde,Alkene, Carboxylic Acid, Carboxylic Acid Ester, Dialkylether, Ketone, Methyl, Phenol, Primary Amine, Primary Alcohol, Secondary Alcohol, Secondary Amine, Tertiary Alcohol (64.03%)
Distinct Functional Group + Subgraph	Ketone, Methyl, Secondary Alcohol (51.75%)	Dialkylether, Enol, Ketone, Methyl, Secondary Alcohol (57.03%)	Dialkylether, Enol, Enolether, Ketone, Methyl ,Phenol, Primary Alcohol, Secondary Alcohol, Secondary Amine, Tertiary Alcohol (63.33%)	1,2-diol, Aldehyde, Carboxylic Acid, Carboxylic Acid Ester, Enol, Enolether, Ketone, Methyl ,Phenol ,Primary Amine, Primary Alcohol, Secondary Alcohol, Secondary Amine, Tertiary Alcohol, Tertiary Amine (65.85%)
Distinct Functional Group + Overlapping	Alkene, Ketone, Secondary Alcohol (51.8%)	Alkene, Carboxylic Acid, Ketone, Methyl, Secondary Alcohol (56.66%)	Alkene, Alkylarylethermol, Carboxylic Acid, Carboxylic Acid Ester, Ketone, Methyl, Phenol, Primary Alcohol, Secondary Alcohol, Tertiary Alcohol (62.51%)	5-Heterocycle, Aldehyde, Alkene, Alkylarylethermol, Carboxylic Acid, Carboxylic Acid Ester, Dialkylether, Enamine, Ketone, Methyl, Phenol, Primary Alcohol, Secondary Alcohol, Secondary Amine, Tertiary Alcohol (65.19%)
Distinct Functional Group + Overlapping + Subgraph	Dialkylether, Ketone, Secondary Alcohol (51.32%)	Dialkylether, Enol, Ketone, Methyl, Secondary Alcohol (56.51%)	5-Heterocycle, Carboxylic Acid, Enol, Enolether, Ketone, Methyl, Primary Alcohol, Secondary Alcohol, Secondary Amine, Tertiary Alcohol (62.73%)	1,2-diol, 5-Heterocycle, 6-Heterocycle, Aldehyde, Carboxylic Acid, Carboxylic Acid Ester, Enol, Enolether, Ketone, Methyl, Primary Alcohol, Secondary Alcohol, Secondary Amine, Tertiary Alcohol, Tertiary Amine (65.13%)
Distinct Functional Group + Overlapping + Subgraph + Super	Ketone ,Super Carboxylic Acid Derivative, Super Hydroxyl (52.03%)	Ketone, Methyl, Secondary Alcohol, Super Carboxylic Acid Derivative, Super Ether (56.84%)	Enol, Enolether, Ketone, Methyl, Primary Alcohol, Secondary Alcohol, Secondary Amine, Super Carboxylic Acid Derivative, Super Ether, Tertiary Alcohol (63.27%)	1,2-diol, 5-Heterocycle, 6-Heterocycle, Aldehyde, Enamine, Enol, Enolether, Ketone, Methyl, Primary Alcohol, Secondary Alcohol, Secondary Amine, Super Carboxylic Acid Derivative, Super Ether, Tertiary Alcohol (65.42%)
Super Only	Super Carboxylic Acid Derivative, Super Ether, Super Hydroxyl (51.18%)	Super Amine,Super Carboxylic Acid Amide,Super Carboxylic Acid Derivative,Super Ether,Super Hydroxyl (52.79%)	Algorithm terminated due to performance cutoff	Algorithm terminated due to performance cutoff

Table S6 C) The optimal pseudostoichiometric strategies provide nearly the same percent of disambiguation as the stoichiometric strategies but with slightly different functional groups comprising each strategy until the number of functional groups within a strategy becomes larger than five. Pseudostoichiometric adduct formation provides much better performance than non-stoichiometric and is an achievable goal for CS-tagging strategies.

Table S6 C: Functional groups comprising best non-stoichiometric strategies for KEGG only

Strategy:	Top 3	Top 5	Top 10	Top 15
Distinct Functional Group Only	Alkene, Ketone, Methyl (59.32%)	Alkene, Carboxylic Acid, Ketone, Methyl, Secondary Alcohol (63.33%)	Aldehyde, Alkene, Carboxylic Acid, Ketone, Methyl, Phenol, Primary Alcohol, Secondary Alcohol, Secondary Amine, Tertiary Alcohol (66.82%)	1,2-diol, 1,2-diphenol, Aldehyde, Alkene, Carboxylic Acid, Carboxylic Acid Ester, Dialkylether, Ketone, Methyl, Phenol, Primary Amine, Primary Alcohol, Secondary Alcohol, Secondary Amine, Tertiary Alcohol (68.13%)
Distinct Functional Group + Subgraph	Dialkylether, Methyl, Secondary Alcohol (58.43%)	Dialkylether, Enol, Ketone, Methyl, Secondary Alcohol (63.42%)	Alkene, Dialkylether, Enol, Enolether, Ketone, Methyl, Phenol, Primary Alcohol, Secondary Alcohol, Secondary Amine (68.88%)	1,2-diol, Aldehyde, Alkene, Dialkylether, Enol, Enolether, Ketone, Methyl, Phenol, Primary Amine, Primary Alcohol, Secondary Alcohol, Secondary Amine, Tertiary Alcohol, Tertiary Amine (70.65%)
Distinct Functional Group + Overlapping	Alkene, Methyl, Secondary Alcohol (59.43%)	Alkene, Ketone, Methyl, Phenol, Secondary Alcohol (64.09%)	5-Heterocycle, Aldehyde, Alkene, Carboxylic Acid, Ketone, Methyl, Phenol, Primary Alcohol, Secondary Alcohol, Secondary Amine (68.15%)	1,2-diphenol, 5-Heterocycle, Aldehyde, Alkene, Alkylarylethermol, Alpha-aminoacid, Carboxylic Acid, Enamine, Ketone, Methyl, Phenol, Primary Alcohol, Secondary Alcohol, Secondary Amine, Tertiary Alcohol (69.72%)
Distinct Functional Group + Overlapping + Subgraph	Dialkylether, Methyl, Secondary Alcohol (58.1%)	Dialkylether, Enol, Ketone, Methyl, Secondary Alcohol (63.59%)	1,2-diol, Alkene, Dialkylether, Enol, Enolether, Ketone, Methyl, Primary Alcohol, Secondary Alcohol, Secondary Amine (68.59%)	1,2-diol, 6-Heterocycle, Aldehyde, Alkene, Alpha-aminoacid, Dialkylether, Enol, Enolether, Ketone, Methyl, Phenol, Primary Alcohol, Secondary Alcohol, Secondary Amine, Tertiary Alcohol (70.27%)
Distinct Functional Group + Overlapping + Subgraph + Super	Methyl, Super Carboxylic Acid Derivative, Super Hydroxyl (58.99%)	Enol, Methyl, Secondary Alcohol, Super Carboxylic Acid Derivative, Super Ether (63.8%)	1,2-diol, Alkene, Enol, Enolether, Ketone, Methyl, Secondary Alcohol, Secondary Amine, Super Carboxylic Acid Derivative, Super Hydroxyl (68.89%)	1,2-diol, 1,2-diphenol, 6-Heterocycle, Alkene, Alpha-aminoacid, Enamine, Enol, Enolether, Ketone, Methyl, Primary Alcohol, Secondary Alcohol, Secondary Amine, Super Carboxylic Acid Derivative, Super Hydroxyl (70.56%)
Super Only	Super Carboxylic Acid Derivative, Super Ether, Super Hydroxyl (55.9%)	Super Amine, Super Carboxylic Acid Amide, Super Carboxylic Acid Derivative, Super Ether, Super Hydroxyl (57.11%)	Algorithm terminated due to performance cutoff	Algorithm terminated due to performance cutoff

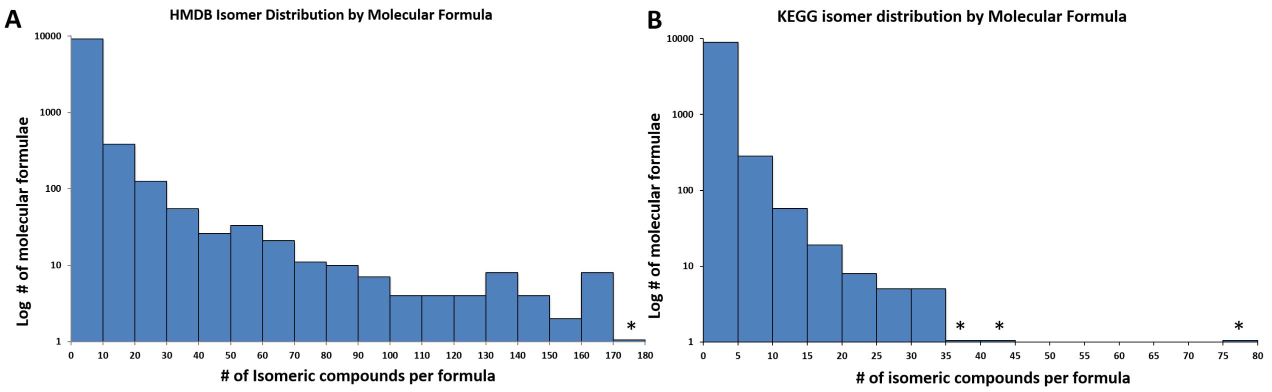

Figure S1: Isomeric compound distribution by molecular formula within the HMDB and KEGG. Within each database, all isomeric compounds and their respective formulas were determined. The number of isomeric compounds mapping to each formula was then calculated; the number of formulas with a specific number of isomeric compounds was then determined and plotted (e.g. two formulas have 27 isomeric compounds). Bins marked * represent bins with one molecular formula, these could not be plotted due to the log transform. **A)** In the HMDB, a large number of formulas have over 40 compounds that map to them and a significant portion have over 100 compounds. Since the HMDB contains many similar entries that are structural isomers of one another, many of these compounds that map to the same formula are very similar in bonded structure, making disambiguation of all compounds mapping to the formula very difficult. (e.g. the many lipids in the HMDB) **B)** The distribution of isomers in KEGG differs significantly from the HMDB. In KEGG, all but three formulae have 35 or fewer compounds that map to them. Isomers in KEGG are mapped to a relatively larger number of formulae; making it easier to disambiguate them. Additionally, KEGG does not have as many lipid entries as the HMDB, reducing a source of very difficult compounds to disambiguate.

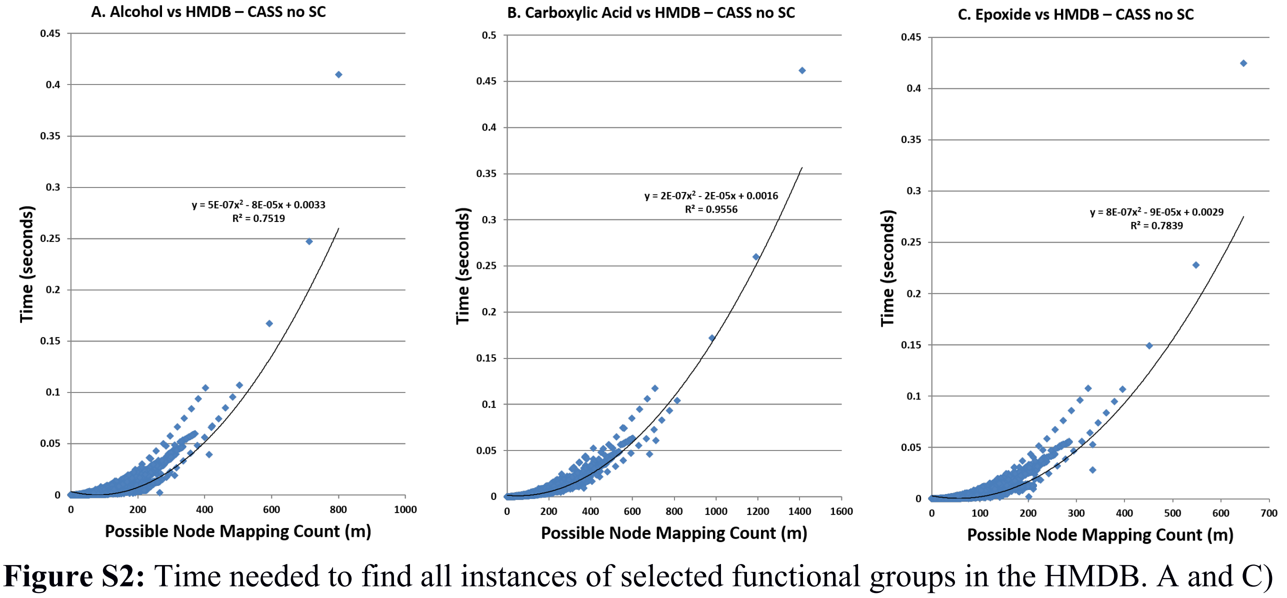

Figure S2: Time needed to find all instances of selected functional groups in the HMDB. A and C) Unlike the time needed to find all instances of alkenes in the HMDB, which was neatly polynomial, the R^2 values for figures A and C show that there is some deviation from the behavior observed when searching for alkenes. Unlike the alkene group which contains only carbon, there are multiple element types which must be matched by CASS in order to find a valid instance of the functional group. When multiple element types are considered, there are now separate search spaces for each element type within the functional group as each element type in the functional group is only tested against the same element type in the database compound when wild card atoms are not considered. Since certain element types are more common than others, the sizes of these search spaces differ and if it is determined that there is no valid mapping for one element type, the algorithm terminates

as a valid mapping of the whole functional group is impossible. Therefore, we see two polynomial curves in all three of the trials for these functional groups. Although both alcohols and carboxylic acid contain hydrogens as well as oxygens and carbons, essentially there is always at least one valid hydrogen mapping given the ubiquity of hydrogen in the metabolome. Due to this effect, the scatter plots are not fit well by a *single* polynomial fit line, resulting in smaller R^2 values. B) Although the same effect occurs when searching for carboxylic acid functional groups, the larger values of m minimize this effect.
